# Bet Hedging in Yeast by Heterogeneous, Age-Correlated Expression of a Stress Protectant

**DOI:** 10.1371/journal.pbio.1001325

**Published:** 2012-05-08

**Authors:** Sasha F. Levy, Naomi Ziv, Mark L. Siegal

**Affiliations:** Center for Genomics and Systems Biology, Department of Biology, New York University, New York, New York, United States of America; University of Bath, United Kingdom

## Abstract

Genetically identical cells grown in the same culture display striking cell-to-cell heterogeneity in gene expression and other traits. A crucial challenge is to understand how much of this heterogeneity reflects the noise tolerance of a robust system and how much serves a biological function. In bacteria, stochastic gene expression results in cell-to-cell heterogeneity that might serve as a bet-hedging mechanism, allowing a few cells to survive through an antimicrobial treatment while others perish. Despite its clinical importance, the molecular mechanisms underlying bet hedging remain unclear. Here, we investigate the mechanisms of bet hedging in *Saccharomyces cerevisiae* using a new high-throughput microscopy assay that monitors variable protein expression, morphology, growth rate, and survival outcomes of tens of thousands of yeast microcolonies simultaneously. We find that clonal populations display broad distributions of growth rates and that slow growth predicts resistance to heat killing in a probabalistic manner. We identify several gene products that are likely to play a role in bet hedging and confirm that Tsl1, a trehalose-synthesis regulator, is an important component of this resistance. Tsl1 abundance correlates with growth rate and replicative age and predicts survival. Our results suggest that yeast bet hedging results from multiple epigenetic growth states determined by a combination of stochastic and deterministic factors.

## Abstract 2

A new experimental approach reveals a bet-hedging strategy in unstressed, clonal yeast cells, whereby they adopt a range of growth states that correlate with expression of a trehalose-synthesis regulator and predict resistance to future stress.

## Author Summary

Genetically identical cells grown in the same environment can display heterogeneity in their morphology, behavior, and composition of their cellular components. In some microorganisms, such cellular heterogeneity can underlie a phenomenon known as bet hedging because it enables some cells to survive in harsh environments, hence increasing the overall population fitness when environmental shifts are unpredictable. Bet hedging is likely to be an important strategy by which microbes infect humans and evade antimicrobial treatments, yet little is known of how cellular heterogeneity contributes to microbial survival. Here, we study the mechanisms underlying bet hedging in yeast. We find that populations of genetically identical yeast contain a broad distribution of growth rates and that slow growth predicts resistance to heat killing in a graded fashion. We identify several gene products that are likely to play a role in this bet-hedging strategy and confirm that Tsl1, a regulator of the production of the disaccharide trehalose, is an important component of acute stress resistance. Finally, we find that old age in cells correlates with a Tsl1-abundant, stress-resistant cell state. Our results suggest that trehalose synthesis is part of a complex and multifactorial mechanism that underlies bet hedging in yeast.

## Introduction

Clonal populations of cells grown in a constant environment display a striking amount of cell-to-cell heterogeneity. For example, in bacteria, yeast, and mammalian cell lines, levels of some gene products vary widely between cells [1]–[5]. A crucial challenge is to understand how much of this heterogeneity serves a biological function [6],[7]. That is, does variability in gene expression between clonal cells simply reflect the noise tolerance of a robust system, or does the variation itself increase population fitness?

In bacteria, several examples exist in which clonal variation in gene expression correlates with a morphological or physiological state that presumably confers a fitness advantage in some environments. These examples include competence to uptake foreign DNA [8]–[10], initiation of sporulation [11],[12], and expression of cell surface pili [13],[14]. In each case, a binary fate decision is controlled in part by stochastic expression of a crucial regulatory protein.

A population-fitness advantage for heterogeneity is even more obvious for the phenomenon known as bacterial persistence. When a clonal population of *Escherichia coli* is exposed to a lethal dose of ampicillin, the vast majority of the population dies at a fast exponential-decay rate but rare, slow-growing “persister” cells die at a much slower rate [15],[16]. These persister cells can subsequently switch to the common, fast-dividing state, thereby restoring the population after removal of the antibiotic. Persistence is therefore considered a canonical example of a bet-hedging mechanism (Box 1), whereby a population maximizes its long-term fitness in an unpredictably changing environment by distributing risk among individuals [16],[17]. In a benign environment, most *E. coli* cells adopt the sensible strategy of fast growth, whereas a small proportion of cells adopt the high-risk strategy of entering the persister state, which could reap large benefits should the environment change.

**Box 1. Bet Hedging: Definitions and Open Questions**

Bet hedging is an often loosely used term to describe a risk-spreading strategy that increases a population's fitness in unpredictably fluctuating environments. A rigorous definition of bet hedging is reversible epigenetic phenotypic heterogeneity that results in decreased arithmetic mean fitness and increased geometric mean fitness of the population across environments [87]. This concept can be best understood in microbes that compete in a benign environment most of the time but unpredictably encounter a harsh environment. In the common benign environment, a heterogeneous population will be less fit than one with a single robust phenotype tuned to the benign environment. However, when acute transitions to the harsh environment occur, the heterogeneous population will contain some individuals better able to cope with it, and thereby will outcompete the robust population.

The requirements for an experimental demonstration of bet hedging are currently undergoing a lively debate [88]. General agreement underlies several criteria: (1) bet hedging is epigenetic in nature and therefore must be demonstrated in isogenic lines; (2) bet hedging must be demonstrated to act across unpredictable environmental shifts where it could be reasonably assumed that a sense-and-response system would have greater costs than benefits [17]; (3) cell lineages must be demonstrated to interconvert between phenotypic states reversibly and independently of the prevailing environment; and (4) alternative phenotypic states must be demonstrated to confer different fitnesses across environments.

Several other proposed requirements for bet hedging lack consensus. The first concerns whether or not interconverting phenotypic states must be binary. Early examples of putative bet-hedging systems in bacteria meet this criterion [8]–[16]. For example, a discrete slow-growth phenotype in *E. coli* predicts survival of high doses of ampicillin [15],[16]. However, several examples in yeast demonstrate multiple metastable phenotypic states [41]–[43], and recent observations of macroscopic bacterial colonies on agar have found a continuous distribution of growth rates [44]. Heretofore, it remained an open question whether multiple discrete phenotypes or a continuous distribution of phenotypes could act as a risk-spreading mechanism.

A second debate surrounds the mechanism by which cells interconvert between phenotypic states. Because phenotypic switching must be insensitive to the environment, it has been generally assumed that the mechanism underlying switching must be stochastic [16],[17],[89]. We argue here that deterministic factors, such as replicative age, could also underlie bet hedging. For example, unequal segregation of certain molecular components between mother and daughter cells [35]–[38] or daughter-specific expression [39] could produce meaningful replicative-age-dependent fitness differences within a yeast population that is independent of environmental shifts [24]. Indeed, a completely deterministic asymmetric cell division has been found to underlie fitness differences under starvation in the bacterium *S. meliloti* [45].

A third debate surrounds the selective forces that ultimately produce a bet-hedging system. That is, if a distribution of phenotypes and relative fitnesses is produced as a by-product of some other adaptive event, can this be classified as a bet-hedging system? Or, must selection be for the distributed phenotypes per se? For example, old cells might be selected to increase production of chaperones to compensate for an increased misfolded-protein load. A by-product of the increased abundance of chaperones might be an increased heat tolerance of old cells and the observation of survival heterogeneity within a population exposed to acute heat stress. To demonstrate conclusively that survival heterogeneity is a consequence of selection for distributed phenotypes, however, the environmental regime and fitness distributions must be measured during the adaptation itself [45]. This represents an extreme experimental challenge that has been overcome only a few times [89]–[91]. Indeed, in a comprehensive survey of over 100 studies of candidate bet-hedging systems, Simons [92] found only four cases meeting his most stringent evidence criteria. We therefore propose to reserve the term “adaptive bet hedging” [93],[94] for cases where survival heterogeneity has been demonstrated to be consequence of selection for distributed phenotypes and use the term “bet hedging” in all other cases where the consensus criteria have been met. In the present study, we show reversible and environment-independent interconversion of phenotypic states that results in heterogeneity of survival for isogenic cells exposed to acute stress. We therefore refer to this phenomenon as bet hedging, while acknowledging that the hypothesis of its being adaptive remains to be tested.

Single-cell observations in a microfluidic chamber suggest that, as with competence and the other examples above, persisters and non-persisters constitute binary states that interconvert through a stochastic mechanism [16]. However, despite the clinical importance of persistence, and despite indications that slow growth might be a general means of surviving stress [18]–[20], the molecular mechanisms underlying persistence remain unclear [21],[22]. This is due, in large part, to the experimental difficulty of identifying and characterizing a rare persister subpopulation prior to antimicrobial treatment.

Like clonal populations of bacteria, those of the budding yeast *S. cerevisiae* have also been shown to contain a large amount of cell-to-cell heterogeneity [3],[4],[23],[24]. In both bacteria and yeast, one component of gene-expression variation is so-called “intrinsic” noise, which is operationally defined as fluctuations that are not correlated between identical promoters in the same cell [3]. In yeast, as in other eukaryotes, an important component of intrinsic noise is fluctuation between more or less accessible chromatin states [3],[5],[25]–[28]. Mutations in yeast genes associated with chromatin remodeling alter the extent of heterogeneity in both protein expression [3],[29] and cell morphology [30].

By contrast, “extrinsic” noise is defined as variation that is correlated between different alleles of the same gene, or between different genes [3]. Such variation reflects either fluctuations in the concentrations of upstream regulators (i.e., intrinsic noise upstream can produce extrinsic noise downstream), or fluctuations in global cell state, such as the abundances of ribosomes [31] or mitochondria [32].

In yeast, evidence suggests that a fraction of what might operationally be defined as extrinsic noise is instead due to deterministic factors. For example, fluctuations of many gene products have been found to correlate with the cell cycle [4] and cell size [3],[33],[34]. Additionally, unequal segregation of certain molecular components between mother and daughter cells [35]–[38] or daughter-specific expression [39] could produce meaningful replicative-age-dependent heterogeneity within a yeast population [24]. For example, cells that have undergone ∼eight replicative cycles survive ultraviolet irradiation better than younger or older cells [40].

A combination of stochastic and deterministic influences could provide the basis for more complex bet-hedging mechanisms than the binary switches that appear to be primary in bacteria. Indeed, the pathogenic yeast *Candida albicans* displays at least seven different metastable colony morphologies when grown on agar [41]. Another opportunistic pathogen, *C. glabrata*, which despite its name is actually a member of the *Saccharomyces* clade, shows similar multi-stability [42],[43]. It should also be kept in mind that bacterial bet-hedging mechanisms might be more complex as well, and that the apparent primacy of binary switches might be a product of the phenotypes chosen for study and of experimental limitations in phenotypic measurement. For example, although *E. coli* antibiotic persistence is commonly described as a two-state system, recent observations of macroscopic bacterial colonies on agar have found a continuous distribution of growth rates [44]. Additionally, asymmetric cell division has been found to underlie bet hedging to starvation in the bacterium *Sinorhizobium meliloti*, indicating that deterministic factors may be important in prokaryotes as well [45].

Here, we investigate the mechanisms of bet hedging and persistence in *S. cerevisiae* using a new high-throughput microscopy assay capable of monitoring variable protein expression, morphology, growth rate, and survival outcomes of tens of thousands of yeast microcolonies simultaneously. We find that clonal populations of yeast grown in a rich, benign environment display a wide and continuous distribution of growth rates that can be modulated by mutations in genes involved in chromosome organization or other core regulatory functions. Using a bioinformatic screen, we identify candidate gene products whose expression correlates with growth rate and establish that Tsl1, a protein involved in the synthesis of the disaccharide trehalose, is a molecular marker for slow growth in the benign environment. Using quantitative measurements of microcolony growth rates and abundance of fluorescently tagged Tsl1, we show that both slow growth and Tsl1 abundance predict survival of heat stress in a graded rather than binary fashion and that Tsl1 is an important component of the stress survival. Lastly, we investigate replicative age as a potential source of heterogeneity in this stress-survival system and in protein expression in general. We find that Tsl1-abundant cells tend to be older and, more generally, that replicative age is an underlying component of cell-to-cell variation in the expression of many proteins.

## Results

### High-Throughput Microcolony Growth Assay

Microbial fitness assays have historically been limited to ensemble measurements that calculate the difference in mean growth rate or the competitive fitness advantage of one population over another. Besides suffering severe limitations in the number of replications that are experimentally feasible, these assays do not measure the variance of growth rates, even though this is likely to be an evolutionarily meaningful parameter in both static and fluctuating environments and over the course of population bottlenecks [17],[24],[46]–[49].

**Figure 1:**
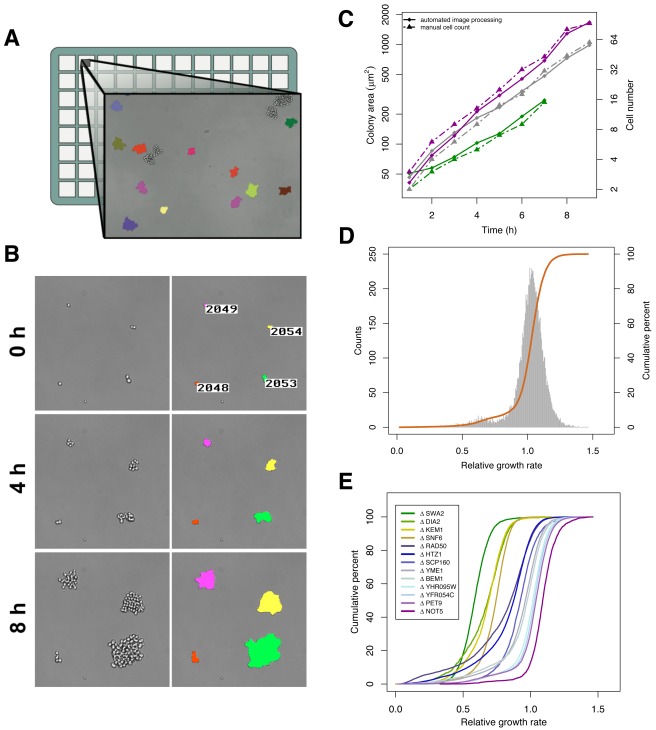
A new high-throughput assay to measure growth rate variance in yeast. (A) A schematic of the assay. Cells in logarithmic growth are plated at a low density on a glass-bottomed multi-well plate. Low-magnification time-lapse bright-field images are captured in a highly parallelized manner. Custom-written software tracks colony area over time. (B) Isogenic cells grow at different rates. Time-lapse images of a portion of one field (left) and the output of image analysis software (right). (C) Microcolony area correlates with cell number. Representative traces of fast- (purple), medium- (grey), and slow- (green) growing microcolonies from a strain of the yeast deletion collection containing the knockout of *YFR054C*, an open reading frame with dubious function. Colony area as determined by automated image processing (solid lines, diamonds) and cell number as determined by a manual count (dashed lines, triangles) are plotted over time. Colonies generally display log-linear growth over the duration of the experiment. (D) Isogenic cells display a wide distribution of growth rates. A histogram of growth rates (grey) and a cumulative growth rate distribution (orange) of a population of ∼17,000 isogenic microcolonies of the *YFR054C* knockout. (E) Gene deletions alter both growth rate mean and variance. Cumulative growth rate distributions of strains from the yeast deletion collection: two knockouts of open reading frames with dubious function (*YFR054C* and *YHR095W*), two knockouts that are unable to grow without mitochondrial function (petite-negatives *PET9* and *YME1*), and nine knockouts with diverse functions that have been shown to result in cell-to-cell heterogeneity in protein expression or morphology (*SWA2*, *DIA2*, *KEM1*, *SNF6*, *RAD50*, *HTZ1*, *SCP160*, *BEM1*, and *NOT5*). Note that a steep slope for the cumulative growth rate distribution indicates a low variance in growth rate.

To overcome these limitations, we developed a high-throughput assay that measures microcolony growth by time-lapse bright-field microscopy (Figure 1A; Videos S1 and S2). Exponentially growing cells are plated at a low density in rich, liquid medium on glass-bottomed micro-well plates and allowed to grow into isolated microcolonies of up to ∼100 cells (Materials and Methods). During this growth period, 1-h time-lapse images of ∼3,000 low-magnification fields are captured in parallel allowing for simultaneous observation of ∼10^5^ microcolonies. Custom-written image analysis software tracks changes in area over time, and these measurements are used to calculate the specific growth rate of each microcolony (the change in the log of the area per hour). Each growing microcolony displays log-linear growth over the period of observation (Materials and Methods), yet different microcolonies grow at vastly different rates (Figure 1B and 1C). The automated measurements of microcolony area correlate extremely well with manual cell counts over a range of growth rates (*R* ^2^>0.9) (Figures 1C, S1, and S2), indicating that changes in area are representative of cell-division rates. Growth-rate distributions generated from all individual microcolony growth rates measured within a well of a 96-well plate are highly reproducible between wells on a single plate or between experimental days (Figure S3).

In wild-type populations grown in a benign environment, a large fraction of microcolonies grow at less than half the median population growth rate (1.3%–10%, depending on the strain) (Figure 1D; Table S1). Because growth rate is extremely consistent within a microcolony over the duration of tracking, this wide distribution indicates that substantial differences in growth rate between isogenic cells exist and are heritable over several generations. We hypothesized that, as in bacteria, cell growth rates constitute a phenotypically observable component of epigenetic cell states that together act as a bet-hedging mechanism in yeast. That is, the lowered relative fitness of slow-growing cells in the benign environment would have an increased relative fitness in other, perhaps harsher, environments, allowing a clonal population to maximize the population fitness over multiple environments. We first ruled out several alternative technical and biological explanations of slow growth. One possibility is that local nutrient depletion by neighboring microcolonies causes closely spaced microcolonies to grow slower than distantly spaced microcolonies. With the exception of microcolonies within 35 µm (4–8 cell lengths) of each other, microcolony growth rate distributions showed no observable dependence on the proximity of a microcolony to its nearest neighbor (Figure S4). A slight difference in growth-rate distribution of microcolonies that fall within 35 µm of each other could be detected and is likely due to a technical bias of the experiment rather than local nutrient depletion (Materials and Methods). Regardless of the cause, removing closely spaced microcolonies had a minimal effect on observed growth rate distributions. Nonetheless, to be conservative, we ignored these microcolonies in all data reported here.

A second possible explanation for the frequent occurrence of slow-growing microcolonies is that these cells are petites, having lost mitochondrial function. Such losses can occur frequently in yeast [50]. To test this possibility, we generated growth rate distributions of single-deletion strains of several genes necessary for growth as a petite [50],[51] and compared these to growth distributions of control strains of the same genetic background but with a deletion of a dubious open reading frame (Figures 1E and S5). Petite-negative strains generally contain as many or more slow-dividing microcolonies than controls, suggesting petites are not a major component of slow-dividing microcolonies in our assay.

Lastly, we considered a high mutation rate as a possible source of slow growth. Based on mutation accumulation experiments, the spontaneous mutation rate in *S. cerevisiae* is estimated to be ∼0.003–0.006 per cell per DNA replication when mutations to homopolymeric runs are excluded [52],[53], and ∼0.3 when mutations to homopolymeric runs are included [52]. Assuming that each mutation results in an observable difference in growth rate in our assay, mutation rates of this magnitude could explain a large fraction of the growth rate variation. However, the deleterious mutation rate is expected to be far lower than the spontaneous mutation rate, and orders of magnitude below the number of slow-growing colonies we observe. Indeed, in *S. cerevisiae* the rate of fitness-altering mutations has been estimated to be 1.37×10^−4^ per haploid genome per generation [54]. Moreover, we show below that slow growth is reversible for both single cells and cell populations, suggesting that a large component of growth rate heterogeneity is metastable and epigenetic in nature.

### Mutations Alter the Variance in Growth Rate

We have shown that wild-type yeast populations grown in a benign environment contain a wide distribution of growth rates, a property likely to impact population fitness in both static and fluctuating environments. A static environment favors low variance in growth rate, as the long-term population growth rate of a single genotype is its geometric mean, which weighs lower values of a distribution more heavily [55]. In contrast, a fluctuating environment can favor high variance, if growth rate correlates with stress survival [17]. The variance of the growth-rate distribution is therefore an important evolutionary parameter, but one that is invisible to standard, population-level measurements of growth rate. To examine whether mutations can alter the variance in growth rate, we selected candidates from previous studies that had shown that deletions in gene products involved in chromosome organization or those with a large number of genetic and physical interactions increase the cell-to-cell heterogeneity in gene expression or morphology [3],[29],[30],[56]. We find that the variance in growth rate can also be modulated by these deletions (Figure 1E; Table S1). For example, deletion of histone variant *HTZ1* or the protein scaffold *BEM1* results in a greater than 4-fold increase in slow-growing microcolonies (operationally defined as microcolonies growing at less than half of the population median) when compared to control deletions of dubious open reading frames. Interestingly, some gene deletions decrease the growth rate variance. For example, deletion of *SWA2*, a gene that encodes a product involved in clathrin-dependent vesicular transport, and *NOT5*, a gene that encodes a global transcriptional regulator, each result in a greater than 2-fold decrease in the number of slow-growing microcolonies. There does not appear to be a trivial relationship between the mean growth rate and variance (Figure S6). A deletion resulting in a reduced mean growth rate can result in an increased (*DIA2*, *RAD50*, *HTZ1*, *BEM1*) or decreased (*SWA2*, *SNF6*) variance when compared to a deletion of a dubious open reading frame.

### Tsl1 Is a Marker for Slow Growth in a Benign Environment

**Figure 2:**
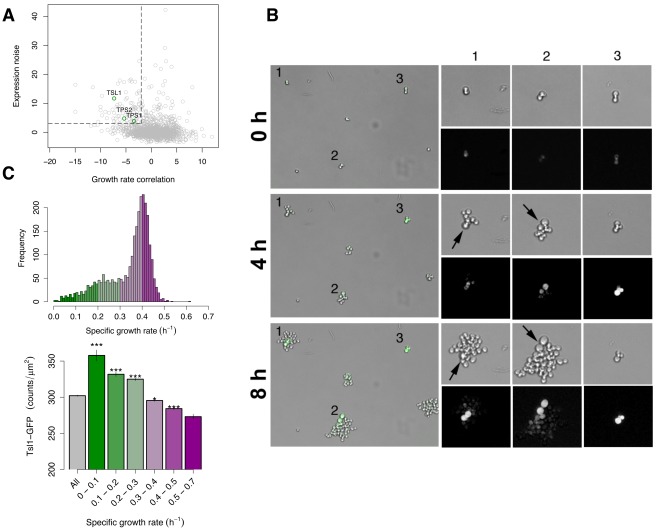
Tsl1 protein content marks slow growth. (A) A bioinformatic screen for candidates marking slow-growing cells. The correlation of mRNA expression with the bulk population growth rate [18] is plotted against the protein-expression noise (the extent of cell-to-cell variation in expression, DM in synthetic dextrose media from [4]) for each gene. 78 noisy genes that anti-correlate with the growth rate are in the upper left quadrant (dashed lines). Among these are three subunits of the trimeric trehalose synthase complex (green circles). (B) Time-lapse microscopy of cells expressing Tsl1-GFP under the endogenous *TSL1* promoter. Three time points of Tsl1-GFP fluorescence overlaid onto bright-field (left) and 1.67-fold magnified views of bright-field (top right for each time point) and GFP fluorescence (bottom right for each time point) of three colonies from the field. Arrows indicate the emergence of a morphologically distinct, slowly dividing, Tsl1-GFP fluorescent cell within a colony. (C) Tsl1 abundance correlates with growth rate. Top: A histogram of the specific growth rates of *TSL1*-GFP cells. Colors indicate bins used in bottom. Bottom: Tsl1-GFP fluorescence intensity per unit area of colonies binned by growth rate. Error bars indicate standard error of the mean (SEM); *p*-values are a comparison to all colonies; Wilcoxon-Mann-Whitney test: *, *p*<0.01; ***, *p*<1×10^−10^.

To allow for further investigation into the nature of growth heterogeneity, we next sought to identify a molecular marker of slow-dividing cell subpopulations. We reasoned that such a marker would have at least two general characteristics: (1) a correlation between its expression level and growth rate, and (2) high cell-to-cell variation in its expression to match the observed variation in growth rate. Genome-wide expression profiling of cells grown at different growth rates in nutrient-limited chemostats has revealed a large number of genes whose transcript levels correlate with growth rate, no matter what the limiting nutrient [18]. However, a correlation between a gene's average expression level and the bulk growth rate might merely indicate that the gene is part of a generalized stress response. Indeed, genes whose transcript levels correlate with growth rate overlap significantly with those that are induced as part of a general environmental stress response [18]. To identify candidates among these genes that might be relevant to growth heterogeneity under constant, benign conditions, we therefore cross-referenced the growth-correlation data with data on cell-to-cell variation in each protein's abundance, as measured by flow cytometry of cells engineered to encode a GFP fusion protein at the corresponding endogenous gene [4]. Plotting these two measures revealed several gene products that anti-correlate with the population growth rate and that also exhibit a large amount of cell-to-cell variation in protein levels under benign conditions (Figure 2A). Using strict cut-offs for the growth-correlation and protein-variation datasets, we identified 78 candidate markers of cell-to-cell variation in growth rate (Materials and Methods) (Table S2).

We next investigated the candidate markers of cell-to-cell variation in growth rate for enrichment in gene ontology (http://www.geneontology.org) process, function, and component terms (Table S3). As a group, the candidates appear to be involved in energy storage or mobilization. Specifically, candidates are highly enriched for mitochondrial genes in the proton-transporting ATP synthase complex (*p*<9×10^−5^) and genes involved in the metabolism of the disaccharide trehalose (*p*<0.002). Trehalose is synthesized by a trimeric complex consisting of two enzymatic subunits, Tps1 and Tps2, and one of two interchangeable cofactors, Tps3 and Tsl1 [57],[58]. Among these, Tps1, Tps2, and Tsl1 were identified as candidates in our screen, with Tsl1 ranking especially high for both the growth correlation and protein noise datasets (Figure 2A). As a class, genes involved in trehalose biosynthesis are highly over-represented among those whose expression levels negatively correlate with growth rate and that are induced by heat shock [20]. Both trehalose and Tsl1 appear to be correlated with a stress-resistant cell state in yeast. Expression levels of Tsl1 and bulk trehalose content remain relatively low during exponential growth but rise rapidly as cells reach saturation and become more stress resistant [58],[59]. Trehalose is thought to preserve protein folding under stress [60], and indeed cellular trehalose content correlates with resistance to various forms of stress, including heat, freezing, desiccation, and high ethanol content [60]–[63]. Consistent with a direct role for Tsl1 in stress resistance, deletion of *TSL1* results in increased sensitivity to killing by high ethanol concentrations [61]. Taken together, these data suggest that Tsl1 might not only serve as a marker for a slow-growing, stress-resistant cell state, but might also be an important component of heterogeneity-dependent stress resistance. We therefore chose to focus further examinations on the role of Tsl1 in bet hedging.

To determine if *TSL1* expression correlates with individual slow growth phenotypes in a non-stressful environment, we simultaneously monitored microcolony growth and green fluorescent protein (GFP) fluorescence of cells encoding a Tsl1-GFP fusion protein at the endogenous *TSL1* locus [64]. As mentioned, Tsl1 abundance increases at saturation [58]. To avoid the possibility that variability in exit from stationary phase could confound our results, we maintained cells in logarithmic growth for a minimum of 24 h prior to any measurements. Consistent with previous flow-cytometry data [4], the expression of Tsl1 varies between cells (Figure 2B). An examination of individual microcolonies suggests that, as predicted, Tsl1-GFP fluorescence correlates negatively with cell-division rate. Figure 2B shows that cells undergoing few or no cell divisions over the course of 8 h are highly Tsl1-GFP fluorescent. Although GFP expression level and growth status tend to persist within a cell lineage, they can change. Microcolonies founded by a fast-dividing cell occasionally produce a highly fluorescent cell with a low cell-division rate (Figure 2B). Cells can switch in the opposite direction as well: a highly fluorescent cell with a low cell-division rate can produce low-fluorescence fast-growing progeny (Video S3). In general, slow-dividing cells appear to be larger than fast-dividing cells (Figure 2B), suggesting that these cells might have altered the influence of cell size on the Start transition in late G1 [18],[65].

The connection between high Tsl1-GFP fluorescence and low cell-division rate, which we observe in individual cases such as that shown in Figure 2B, holds as a general trend across many microcolonies tracked in our assay. To control for alterations in Tsl1 abundance that may be caused by differences in cell size, we measured the Tsl1-GFP intensity per unit area of each microcolony (Figures 2C and S7). A negative correlation between Tsl1 abundance and microcolony growth rate is observed across the range of growth rates (Figures 2C and S8), indicating that Tsl1 is a general marker of growth state rather than a marker for only extreme slow growth. Also of note is that the correlation is specific to Tsl1, not a generic property of any protein with variable expression. Expression of the control protein Tma108-GFP, which has a similar average abundance as Tsl1 [66] and is highly variable from cell to cell [4], shows no correlation with microcolony growth rate (Figure S9).

### Slow Growth and Tsl1 Abundance Predict Resistance to Heat Killing

**Figure 3:**
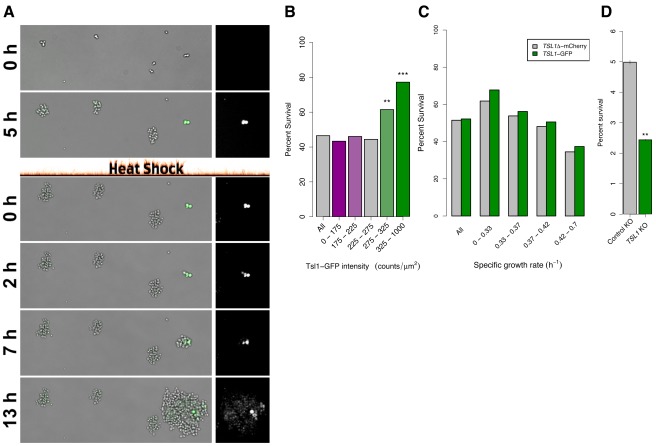
Growth heterogeneity is a stress survival mechanism. (A) Slowly growing, Tsl1 abundant cells survive heat shock. Time-lapse images of Tsl1-GFP fluorescence overlaid onto bright-field (left) and Tsl1-GFP fluorescence of the right-most colony (right) before and after heat shock. Colonies are grown for 5 h to monitor growth rate and Tsl1-GFP fluorescence, heat shocked for 70 min at 60°C, resulting in massive cell death, and monitored for growth for 13 h following heat shock to identify colonies that contain at least one surviving cell. Bright-field and fluorescent images of the entire field at each time point are shown in Video S4. (B) Tsl1-GFP fluorescent colonies are more likely to survive heat shock. Percentage of colonies that contain at least one cell that survives heat shock binned by Tsl1-GFP fluorescence. *p*-Values are a comparison to all colonies, Fisher's exact test: **, *p*<1×10^−5^, ***, *p*<1×10^−10^. (C) *TSL1* contributes to survival in slow-growing colonies. Percentage of colonies that contain at least one cell that survives heat shock binned by growth rate for chimeric *TSL1*-GFP (green) or *TSL1* replaced with mCherry (grey) at the endogenous *TSL1* locus. Both growth rate and *TSL1* genotype significantly affect survivorship (multiple logistic regression, *p*<10^−28^ and *p*<0.01, respectively). (D) *TSL1* contributes to population resistance to acute heat shock. Survival of a strain containing a gene deletion of *TSL1* (green) or a control dubious open reading frame (*YFR054C*, grey) as measured by plating on agar following heat shock of cell suspensions. Student's *t* test of arcsin transformed data; **, *p*<1×10^−4^. Error bars indicate SEM.

Having shown a correlation between Tsl1 abundance and growth rate at the individual microcolony level, we next assayed for differential susceptibility of microcolonies to heat killing. Tsl1-GFP cells were grown normally in our microcolony growth assay for 6 h (producing microcolonies of 1–20 cells), heat shocked under conditions that kill most cells, and placed back under the microscope for an additional 14–20 h of observation (Videos S4 and S5). Figure 3A shows a typical result: a highly fluorescent cell in a slow-growing microcolony survives heat shock, undergoes one or two cell divisions at a slow rate, and then produces fast-growing progeny. Again, this individual case is representative of a general relationship. Microcolonies with a higher Tsl1 content are significantly more likely to contain a survivor, as shown by a plot of the survival frequency of microcolonies binned by the Tsl1-GFP fluorescence prior to heat shock (Materials and Methods) (Figure 3B).

We next asked if Tsl1 is directly involved in heterogeneity-dependent heat resistance or instead acts only as a marker of resistant cells. We generated a genotypically similar *TSL1* knockout strain, by replacing the coding sequence of the Tsl1-GFP fusion protein with that of the fluorophore mCherry, to compare the heat killing susceptibility of the *TSL1*Δ-mCherry strain to the *TSL1*-GFP strain (Figure 3C). Multiple logistic regression was used to isolate the effects of growth rate and *TSL1* genotype on survival (Materials and Methods). Independent of genotype, growth rate before heat shock is a major determinant of survival, with slower growing microcolonies being more likely to contain a survivor (multiple logistic regression, *p*<10^−28^). Because, prior to the heat shock, slow-growing microcolonies produce far fewer cells than do fast-growing microcolonies, the difference in survival per cell is necessarily greater than the differences reported in our microcolony survival assay. In support of a direct role of Tsl1 in heterogeneity-dependent stress resistance, functional Tsl1 improves survival when controlling for growth rate (multiple logistic regression, *p*<0.01) (Figure 3C). The median growth rate of *TSL1*Δ-mCherry populations is slightly reduced compared with *TSL1*-GFP populations (Figure S10) and thus *TSL1*Δ-mCherry populations would be expected to have more survivors if survivorship is independent of *TSL1* content. However, *TSL1*-containing cells are slightly more likely to survive heat killing even without controlling for the effects of growth rate on survival (Figure 3C). One possibility to explain differential survival between the *TSL1*-GFP strain and the *TSL1* deletion is that *TSL1* is an important component of an induced heat shock response rather than a component of a bet-hedging mechanism that renders a proportion of cells heat resistant prior to any environmental shift. To test this possibility, we compared the survival upon extremely acute stress between a *TSL1* knockout from the yeast deletion collection and a second strain from that collection with a deletion of a dubious open reading frame (*YFR054C*). Performing a 2-min heat shock at 60°C in a small volume of liquid medium followed by plating on agar to count survivors (Materials and Methods), we find that *TSL1* directly contributes to heat resistance under these conditions in which an induced response is unlikely to be relevant (Figure 3D).

### Continuous Distributions Underlie Probabilistic Susceptibility to Heat Killing

**Figure 4:**
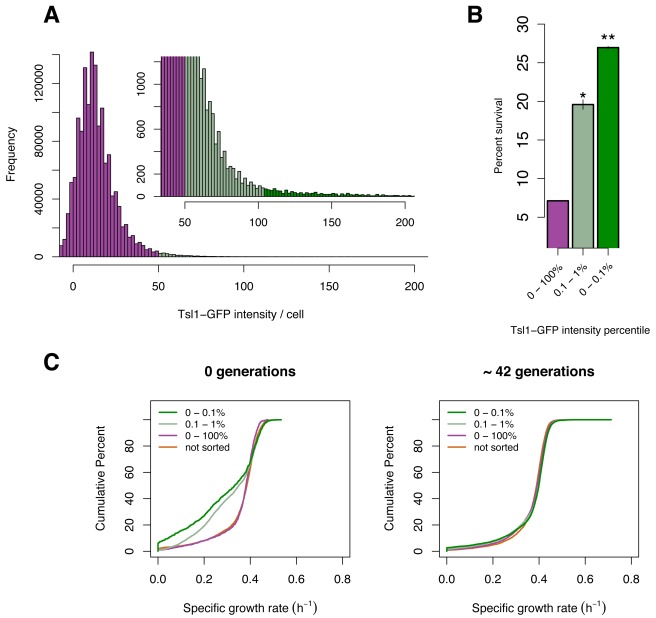
Cell sorting by Tsl1-GFP content alters growth rate distributions and heat shock survival. (A) The distribution of Tsl1 abundance does not appear bimodal. Histogram of single-cell Tsl1-GFP intensity as measured by fluorescence-activated cell sorting. Inset: the right-hand tail of the main figure. Cells with the top 0.1% (dark green) and next 0.1 to 1% (light green) Tsl1-GFP fluorescence were sorted for downstream analysis. (B) Survival of sorted populations following heat shock of a liquid suspension as measured by plating on agar plates. *p*-Values are a comparison to the unsorted population (purple), Student's *t* test of arcsin transformed data: *, *p*<0.01; **, *p*<1×10^−5^. Error bars indicate SEM. (C) Sorting for Tsl1-GFP abundance transiently alters growth rate distributions. Left: cumulative growth rate distributions of sorted cells (dark and light green), cells passed through the cell sorter but unsorted (purple), and cells not passed through the cell sorter (orange). Right: Samples of the same sorted or unsorted cell populations after ∼42 generations of growth.

Having established *TSL1* as both a predictor of susceptibility to heat killing and an important component of the survival machinery, we next sought to characterize the distribution of *TSL1* expression in yeast populations and how this distribution relates to survival. As discussed previously, in bacteria, bistable gene expression patterns underlie several binary phenotypic states thought to act as bet-hedging mechanisms [8]–[14],[24],[67],[68]. Thus, levels of certain proteins show a bimodal distribution across cells. Using flow cytometry to measure cellular Tsl1-GFP fluorescence, we observe a continuous distribution in Tsl1 abundance rather than the bimodal distributions characteristic of bistable bacterial systems (Figure 4A). Sorting cells into discrete bins at the high end of the Tsl1-GFP fluorescence distribution, then subjecting these groups of cells to heat shock reveals that Tsl1 abundance predicts survival in a graded or probabilistic manner rather than a binary manner: the higher the level of Tsl1-GFP, the higher the chance of survival (Figure 4B).

Taken together, observations of continuous or graded distributions in Tsl1 abundance (Figure 4A), growth rate (Figure 2C), and stress survival (Figure 4B) suggest that populations of yeast might contain a continuum of metastable epigenetic cell states that each confer a different fitness in a given environment. This hypothesis is supported by growth-rate distributions derived from cells sorted by Tsl1 abundance. Cells sorted for higher Tsl1-GFP content yield growth-rate distributions containing more slow-growing microcolonies (Figure 4C). If the altered growth-rate distribution of the cells sorted for high Tsl1-GFP fluorescence reflects selection of a subset of metastable cell states, then prolonged culturing of a population founded by the sorted cells should result in a distribution similar to the initial unsorted population, which is presumably a steady-state distribution. The altered growth-rate distribution is indeed transient. After 42 generations of growth, a population founded by sorted cells has a growth-rate distribution that is indistinguishable from that of one founded by unsorted cells (Figure 4C, right).

### Replicative Age Correlates with a Tsl1-Abundant Cell State

**Figure 5:**
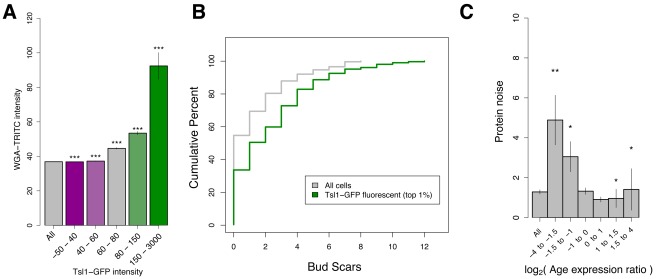
Old cells are Tsl1-GFP abundant. (A) *TSL1*-GFP yeast stained with the bud scar stain WGA-TRITC are passed though a cell sorter to monitor co-fluorescence. Shown is the WGA-TRITC fluorescence of cells binned by Tsl1-GFP fluorescence. *p*-Values are a comparison to all cells, Wilcoxon-Mann-Whitney test: ***, *p*<1×10^−10^. (B) Sorted WGA-TRITC stained *TSL1*-GFP cells are counted for bud scars. Shown is the cumulative percentage of all cells (grey) and cells in the top 1% Tsl1-GFP fluorescence bin (green). The 1% Tsl1-GFP cells have significantly more bud scars, Wilcoxon-Mann-Whitney test, *p*<1×10^−7^. (C) Population demography accounts for some expression “noise.” Shown is the protein-expression noise (DM in yeast permissive dextrose media from [4]) of genes binned by logarithm of their age expression ratio, the average expression in young cells over the average expression in old cells [73]. Error bars indicate SEM; *p*-values are a comparison to all colonies; Wilcoxon-Mann-Whitney test: *, *p*<0.05; **, *p*<0.001.

As discussed previously, a combination of stochastic and deterministic influences is likely to underlie the continuous and graded distributions we observe here. Several characteristics of stress-resistant cells led us to hypothesize that replicative age (the number of cell divisions an individual cell has undergone) could be a deterministic factor underlying yeast bet hedging. For example, both old cells and stress-resistant cells have an increased cell size, altered cellular morphologies, and a slowed cell cycle (Figure 2B) [69]–[71]. To test this hypothesis, we stained *TSL1*-GFP cells with wheat-germ agglutinin (WGA)-tetramethyl rhodamine isothiocyanate (TRITC), a fluorescent marker that specifically stains bud scars, and measured single cell correlations in GFP and TRITC fluorescence by flow cytometry. Older cells show higher levels of TRITC fluorescence because each cell division leaves an additional bud scar [72]. As predicted, cells that abundantly express Tsl1 also show high levels of WGA-TRITC fluorescence (Figure 5A). An alternative explanation is that cell states with high Tsl1 abundance more efficiently take up the WGA-TRITC stain, and the observed correlation is due to differences in staining rather than replicative age. To test this possibility, we sorted cells for high Tsl1-GFP content and compared the number of bud scars in this subpopulation to an unsorted population by performing manual bud scar counts. In further support of an age dependence of Tsl1 expression, we find that cells with abundant Tsl1 tend to have more bud scars (*p*<10^−7^, Wilcoxon-Mann-Whitney test) (Figure 5B).

### Replicative Age Contributes Significantly to Protein-Expression Variation

The finding that variation in replicative age partially underlies heterogeneity in *TSL1* expression (and presumably heterogeneity in growth rate and stress resistance) led us to hypothesize that population demography might underlie a significant fraction of protein-expression variation generally thought to be a consequence of extrinsic noise. To test this hypothesis, we used data from an existing microarray study that measured differences in expression between young (one to three generations) and old (16–18 generations) cells [73]. We then compared these expression differences to data on cell-to-cell variation in each protein's abundance [4]. These abundances were measured by flow cytometry of cells engineered to encode a GFP fusion protein at the corresponding endogenous gene and therefore capture both intrinsic and extrinsic noise, although a major source of extrinsic noise (the cell cycle) was minimized by gating the cells by size and complexity of shape [4]. Plotting the cell-to-cell variation in expression of genes binned by their age expression ratio (AER, the mean expression in young cells divided by the mean expression in old cells) reveals that cell age does indeed contribute significantly to protein-expression variation (Figure 5C, Wilcoxon-Mann-Whitney test). Transcripts that become over- or under-expressed in old cells tend to result in protein levels that are more variable across cells in exponential growth. The absolute log AER explains approximately 1% of the variation in protein-expression variation, which is on par with other significant contributors to protein-expression variation, including mRNA half-life, ribosomal density of mRNA, and translation rate per mRNA [4].

## Discussion

We show that: (1) clonal yeast populations contain a wide and continuous distribution of growth rates when cultured in a benign environment; (2) growth differences are transient and reversible over the course of a few generations; (3) mutations can alter the mean and variance of the steady-state growth rate distribution; (4) Tsl1 is a marker for the slow-growing cells within an exponentially growing population; (5) Tsl1 abundance and slow growth predict resistance to heat killing; (6) *TSL1* is an important component of heterogeneity-dependent heat shock survival; (7) Tsl1-abundant cells tend to be of higher replicative age; and (8) replicative age is likely to underlie a fraction of gene expression heterogeneity for many gene products besides Tsl1.

These results describe a bet-hedging phenomenon in yeast that might be an adaptation to life in an unpredictably varying environment (Box 1). As is true in descriptions of bacterial bet hedging and persistence [16], slow growth is a crucial predictor of stress survival in yeast. Both bacteria and yeast appear to be maximizing population fitness by balancing fast growth in good conditions with bet hedging against bad ones [17].

Yet, some crucial differences between bacterial and yeast bet hedging appear prominent. One difference appears to be in the nature of the heterogeneity underlying bet hedging. Single-cell observations in bacteria suggest that persisters and non-persisters constitute binary growth states that predict survival in an all-or-none fashion [16]. That is, bacterial persisters generally survive and non-persisters generally perish in stress. We find that yeast populations contain a continuous rather than bimodal distribution of growth states and that these states predict survival in a probabilistic manner. That is, the slower a yeast cell grows, the greater its probability of surviving stress. Although the mechanism of bacterial persistence has yet to be elucidated, persisters and non-persisters are thought to interconvert through a stochastic mechanism [16], as is true for the vast majority of characterized bacterial two-state systems [8]–[14]. In yeast, differences in growth and survival appear to be due to a more complex combination of stochastic and deterministic factors. Taken together, these results suggest that bet hedging in yeast is a consequence of a spectrum of metastable inheritable epigenetic states that confer differential fitnesses across environments.

The processes underlying interconversion between epigenetic states, and the different phenotypes associated with these states, are of great importance not just for yeast but also in metazoan development and disease. Interconverting epigenetic states have been shown to underlie phenomena as diverse as antibiotic resistance [16], stem cell reprogramming [74], and cancer progression [75]–[77]. For example, recent work has shown that rare cells within a melanoma tumor divide slowly but give rise to highly proliferative daughter cells, and vice versa [78]. This behavior can be thought of as a bet-hedging mechanism, and likely contributes to the poor long-term performance of chemotherapies that target fast-dividing melanoma cells [78].

Current theoretical models of bet hedging focus on the dynamics of two-state systems [17],[16]. Our results and recent work in human cancer cell lines [77] suggest that future models must account for a distribution of multiple cell states and the transitions between them [79],[80]. Interconversion between multiple Tsl1-abundance and growth states presents an experimentally tractable system that can be exploited to test and parameterize such models. For example, sorting cells by Tsl1 abundance and following changes in growth rate distributions over time might allow for theoretical estimates of the number cell states and the transition rates between them [77]. Additionally, because the microcolony growth and survival assay presented here relies on simple microscopy and image analysis routines, these methods could be relatively easily exported to the above-mentioned cell culture systems to provide additional quantitative measures of metazoan multi-stability and bet hedging.

A correlation between growth and the deterministic factor of replicative age has been previously noted, with increasing age resulting in slower progression through the cell cycle until no more cell cycles can be completed [71]. Here we show that replicative age also correlates with Tsl1-abundant, presumably stress-resistant cell states. We note, however, that replicative age does not appear to be the sole determinant of slow growth, Ts1-abundance, or stress resistance. Both slowed growth and high fluorescence of TSL1-GFP cells persist in newborn cells and their daughters (Figure 1B, 1C; Video S3). Additionally, we observe newly born cells surviving heat shock (Videos S4 and S5). A more likely possibility is that both the deterministic factor of replicative age and stochastic mechanisms contribute to stress resistance, although more research is required to establish causal links.

The possibility that old age contributes to stress resistance provides a particularly compelling bet-hedging mechanism: an old cell with few remaining cell cycles maximizes its contribution to a clonal population if a cell cycle is completed after a stressful event that results in a mass killing of the younger, fast-growing cells. Thus, a slowed cell cycle in old cells—and, with high probability, their few remaining progeny, as implied by inheritance of TSL1 abundance and slow growth from mother to daughter (Video S3)—might be selected to maximize bet hedging. In this scenario, the influence of age, although independent of the environment, could nonetheless be probabilistic, if the age signal or its transduction is noisy. An alternative possibility is that older cells have accumulated minor stresses throughout their lifetimes, so that induction of *TSL1* and other genes represents a genuine stress response despite the benign environment. If true, this possibility would still represent a bet-hedging mechanism, because the induced response protects against a subsequent, unpredictable, and acute stress.

We have previously shown that a large number of gene deletions result in a decreased phenotypic robustness, increasing the cell-to-cell heterogeneity in morphology [30]. Here we show that some of these mutations also alter the growth rate distribution in a benign environment, often resulting in a greater variance in growth rates with proportionally more slow-dividing cells. It is unclear whether the large number of slow-dividing cells in the distributions of these single-deletion strains represent, as they do in wild-type populations, meaningful bets that are more fit in harsh environments, or instead represent unfit cell states in any environment. Yet, the possibility that a large number of mutations could result in increased fitness in harsh environments presents a dynamic picture of the tension between bet hedging and robustness in yeast. That is, selection for a robust phenotype in a given environment (i.e., the fittest phenotypic state) is countered by selection for distributed phenotypes (i.e., multiple phenotypic states that constitute a series of bets on changing environments) [81]. When the environment is not constant and when slow growth in a benign environment confers resistance to an acute stress, then the growth rate distribution of a mutant will be more informative about fitness than the mean growth rate. The high-throughput microcolony assay of growth and stress survival offers a way to explore these distributions systematically using yeast gene-deletion strains or strains segregating natural variation. Another way to explore the tension between robustness and bet hedging would be to test the expectation that organisms evolved in fluctuating environments should exhibit a wider distribution of growth rates than those evolved in static environments.

We have shown here that Tsl1, a protein involved in the synthesis of trehalose, is both a marker and an important component of a stress-resistant cell state. Trehalose appears to function as a general stress protectant across biological kingdoms, approaching 20% of the dry weight of stress-protected organisms such as yeast and nematodes, which regularly encounter harsh conditions [59]. Thus, it is quite plausible that the bet-hedging mechanism described here will provide mechanistic insights into ecological adaptation in a wide range of organisms, as well as into how pathogenic eukaryotes, such as *C. albicans* [82] or indeed strains of *S. cerevisiae* [83], colonize humans and evade therapeutic agents. Identification of Tsl1 as marker for stress-resistant cell states in yeast will be of great value to elucidating the molecular mechanisms underlying persistence, an endeavour that has been elusive in bacterial models [21]. For example, comparisons of the gene expression profiles between cells sorted for abundant Tsl1-GFP and unsorted populations will provide a list of candidate gene products involved in heterogeneity-dependent stress resistance. These candidates can subsequently be tested for correlation to or necessity within a stress-resistant cell state using methods similar to those described here for Tsl1.

## Materials and Methods

### Yeast Strains and Cloning

Haploid deletion strains were converted from the diploid BY4743 YKO magic marker strains (Open Biosystems, MATa/α ura3Δ0/ ura3Δ0 leu2Δ0/leu2Δ0 his3Δ1/ his3Δ1 lys2Δ0/LYS+ met15Δ0/MET15+ can1Δ::LEU2+-MFA1pr-HIS3/CAN1+ xxx::kanMX/XXX+) as described [84]. Tsl1-GFP yeast (MATa his3Δ1 leu2Δ0 met15Δ0 ura3Δ0) are part of the yeast-GFP collection [64] and were purchased from Invitrogen. The *TSL1*Δ-mCherry strain was constructed directly from the *TSL1*-GFP strain by replacing the coding region of the genomic *TSL1*-GFP chimera with that of mCherry and the selectable marker, NatMX, through homologous recombination [85]. The mCherry-NatMX insert was amplified by PCR from the pCZ-Nat plasmid (GenBank accession number JN580989) using the primers (5′→3′) CAAACAAAGCAAAGAATACAATAGCAACGCAAGATCAACACAATGGTGAGCAAGGGCGAGGAGGA and AAGTTCATACCCAAGAAAATTAAAATTTTAAAATGGTAAAATTTATGAGCTCCAGCTTTTGTTCC. The PCR product was extended for homologous recombination using the primers CCGTGTCATTGCACATCCACCCACCCGTCGATTAAAAAACCAAACAAAGCAAAGAATACAATAGCAACG and TAGAATTGATATATAATAAGCAGTTGAAAATAAAAGTTCATACCCAAGAAAATTAAAATTTTAAAATGG. Transformation of the PCR construct was performed with lithium acetate, as described [86], and homologous recombinants were selected for incorporation of NatMX with nourseothricin. Proper integration was confirmed by sequencing.

### Cell Preparation

For all strains, a single colony was selected and grown overnight in YPD to generate a frozen stock. Frozen stocks were struck onto YPD plates at a high density and populations from the streak were used to initiate experiments. Growth rate and survival assays were preformed in synthetic complete liquid medium or on synthetic complete plates. Deletion strains were allowed to reach saturation in liquid culture. A day prior to plating, saturated cell cultures were diluted 1∶60 and grown overnight to saturation. On the day of plating, cultures were again diluted 1∶60 and allowed to reach early logarithmic phase by growing for 3–4 h with shaking at 30°C. Because *TSL1* expression increases for all cells during late log phase and saturation [58], the *TSL1*-GFP and *TSL1*Δ-mCherry strains were instead maintained in early- to mid-log phase for at least 24 h prior to any experiments. We estimate ∼50 generations of growth between a single cell bottleneck and growth rate measurements for single gene deletions strains and ∼60 generations for *TSL1*-GFP and *TSL1*Δ-mCherry strains.

### Microscopy

Cells were sonicated for 90 s on high in a Diagenode Bioruptor water bath, counted using a hemocytometer, and diluted to a final concentration of 5–20×10^3^ cells/ml. Glass-bottomed 96-well plates (Matrical MGB096-1-2-LG) were coated with 200 µl of 200 µg/ml Concanavalin A (Type V, Sigma) for 2–6 h. Wells were washed once with 200 µl of water and 400 µl of cells were plated per well. Plates were sealed with an optically clear film (Axygen PCR-SP) and spun at 360 *g* for 2 min. Before placing the samples on the microscope, the bottom surface of the 96-well plate was dusted using compressed air to remove any particles that may interfere with the Nikon Perfect Focus System (PFS). Micrographs were captured on a Nikon TE2000e microscope equipped with PFS for infrared high-speed focusing and a fully automated stage equipped with a full-stage environmental chamber. All images were collected with a Nikon Plan Apo 10× (0.45 numerical aperture) air objective using Nikon NIS Elements software to drive stage movement and acquisition. Because NIS Elements readily accepts externally written XML files for position and PFS control, we created homemade R- and C-based scripts to assign plate position and focus coordinates that minimize stage travel time and optimize PFS offsets over the plate surface. The environmental chamber was set to 30°C at least 2 h before observation to prevent heat gradients. Prior to image acquisition, a 45-min focusing routine was performed to determine the optimal PFS offset for each well. This focusing routine was necessary because the PFS maintains focus on the plane between the bottom of the cover glass and the air. Thus, alterations in thickness of the cover glass surface result in images that are slightly out of focus, which we found can have mild effects on measured growth rates (unpublished data). Microcolonies begin growing during the focusing routine, thus some microcolonies might contain two cells by the time an initial image is taken. Microcolony growth was monitored by capturing a micrograph of each field every hour. For routines that require only bright field images, we were able to monitor ∼3,000 fields in parallel (∼100,000 microcolonies at 2×10^4^ cells/ml) because each image requires ∼1 s to capture including stage travel time. Tsl1-GFP fluorescence was captured for 4 s at 2× gain. The long exposure time required for fluorescence measurements considerably slowed our assay, allowing us to monitor ∼360 fields (∼3,000 microcolonies at 0.5×10^4^ cells/ml).

### Automated Image Analysis

Image processing and microscope control routines were written in Matlab, R, C, and shell scripts.

#### Focusing routine

For each well, bright-field images of five fields at four different PFS offsets were captured. The five fields map to the center and four corners of a rectangle that covers the central 50% of the area of each well. The four PFS offsets are spaced 7.5 µm apart, which allows at least one image to be captured in the proper focal plane to determine the correct PFS offset (the glass surface typically varies by ∼30 µm over a plate). To find the ideal optical plane for each field in a computationally efficient manner, we took advantage of an idiosyncratic optical property of yeast: ∼10 µm below the ideal focal plane, yeast appear large and white resulting in a histogram of image pixel intensities with a sharp peak at the highest measured pixel intensity. To find the ideal optical plane for each field, we found the PFS offset of the image with maximal number of highest intensity pixels and added 10 µm. For each well, the optimal PFS offset of each of the five fields was averaged and this averaged offset was used for all images captured in the next phase of the experiment. Capturing 1,920 images, (96 wells)×(5 fields)×(4 PFS offsets), requires ∼40 min of microscope time and performing the simple image analysis and calculation routine required about 5 min of computational time.

#### Growth rate analysis

Bright-field images were analyzed continuously during image capture on a different dedicated computer. The area of each micrograph covered by yeast microcolonies was identified by taking advantage of the fact that yeast tend to be both the lightest (high pixel intensities in the cell center and outside the cell perimeter) and dimmest (low pixel intensities at the cell perimeter) objects in the field. Thresholds were applied to both high and low pixel intensities to create a pair of black (not-yeast) and white (yeast) images for each optical field. Each black and white image was then subjected to several rounds of optical dilation and erosion to generate continuous white microcolony objects. Non-yeast objects that were erroneously identified as yeast in either the high or low threshold were removed by performing an AND operation across the high and low pixel threshold images. Thus, only objects that contain both high and low intensity pixels, a property specific to yeast rather than cellular debris or other precipitates, are counted as a microcolony. Once all images from a time-series are collected, microcolonies are aligned through time by centroid proximity. When a microcolony physically touches a neighboring microcolony or the edge of the image field, it is no longer tracked. Large decreases in microcolony area, generally indicating an image analysis failure, cause the software to discontinue tracking that microcolony. Microcolonies that appear de novo in a later time point (generally a single cell that floated away from a nearby microcolony) are grouped with the nearest microcolony if they lie within a distance of (0.65)×(length of the longest line that can be drawn through the microcolony), otherwise they are ignored. For each microcolony, the total recorded time of growth is measured and microcolonies with less than five recorded growth time points are ignored. When log(microcolony area) is plotted over time for 9 h of growth, in excess of 99.9% of microcolonies that underwent at least one cell division (operationally defined as a specific growth rate above 0.1) displayed linear correlations in excess of 0.9, suggesting that cells are not limited for nutrients over the time of observation.

#### Fluorescence quantitation

Tsl1-GFP fluorescence was quantified by averaging the intensities in the fluorescent channel of all pixels within the microcolony area of each microcolony as determined in the above section.

#### Post-heat shock alignment

Removal and replacement of the plate for heat shock causes slight shifts in the locations of each microcolony, often causing errors in centroid-based microcolony alignment through time. Thus, we added a routine to realign microcolonies by searching across many fields on the plate for microcolonies with distant neighbors. The average centroid movement of these isolated microcolonies was used to calculate total plate movement and realign all remaining microcolonies. Reliable realignment required cells to be plated at a lower density. Thus, all heat shock experiments were performed at 5×10^3^ cells/ml (∼eight microcolonies per field). We found that this lower density did not alter growth rate distributions (unpublished data).

#### Microcolony survival

Microcolonies were labeled as survivors if they grew by 400 or more pixels after heat shock (180 µm^2^ or ∼eight cells) in 16 h of growth. Microcolonies were labeled as non-survivors if they grew by less than 300 pixels after heat shock (135 µm^2^ or ∼six cells) in 16 h of growth. Microcolonies that did not meet these criteria were ignored. Alternative values of the change in area cut-offs for calling survivors or non-survivors or of the length of growth allowed before assessing survival had no effect on the conclusions of this paper (unpublished data).

### Measures for Systematic Bias

#### Microcolony proximity measurements

The centroid of each microcolony was calculated by averaging the centroid positions of all time points for which the microcolony was tracked. Proximity of each microcolony to its nearest neighbor was calculated by measuring the minimum distance between a microcolony and all other microcolonies in the field. Microcolonies within 55 µm of the field edge were ignored for all measurements. A small fraction of microcolonies that fall within 35 µm (4–8 cell lengths) of their nearest neighbor do have reduced growth rates (Figure S3). Although it is possible that this skew is due, in part, to local nutrient depletion, a more likely explanation is a technical bias of the experiment: fast-growing close-neighbor microcolonies merge with neighbors before sufficient time points can be recorded to estimate an accurate growth rate (a minimum of five time points is necessary in these assays), whereas slow-growing close-neighbor microcolonies do not.

#### Position on the plate

To measure systematic bias that may be caused by the position of a well on a 96-well plate or the position of a microcolony within a well, we performed several replicates of growth with the identical strain in each well, a haploid segregant of a dubious open reading frame knockout (*YFR054C*) from the BY4743 YKO collection. We found no obvious bias based on well position or position within a well. However, to be conservative, we randomized the well position for all growth rate measurement of strains from the YKO collection.

### Manual Microcolony Cell Counts

Cell counts were performed on a haploid segregant of a dubious open reading frame knockout (*YFR054C*) from the BY4743 YKO collection. Microcolonies on which to perform manual cell counts were selected at random from the following growth rate bins: below two standard deviations (2 SD) from the mean population growth rate, between 2 SD and 1 SD below the mean, between 1 SD below the mean and 1 SD above the mean, between 1 SD and 2 SD above the mean, and above 2 SD above the mean. Counts did not try to distinguish budding cells that have not undergone cytokinesis from two separate cells (i.e., a cell with a small bud was counted as two cells). Microcolonies under ∼100 cells generally grew as a monolayer on the glass surface and cell counts correlated extremely well with automated colony area measurements (Figures 1C, S1, and S2). We did notice that automated measurements slightly overestimated the cell number of the slowest growing microcolonies when they became large (Figure S2A) and slightly underestimated the cell number of the fastest growing microcolonies when they became large (Figure S2E). For both slow and fast growing microcolonies, the automated measurements provide conservative estimates for the deviation from the mean growth rate (i.e., slow-growing colonies are measured as growing faster than their true growth rate), and thus the automated growth rates were not adjusted. For microcolonies over ∼100 cells, we did notice some piling of cells on top of each other resulting in automated colony area measurements underestimating the total number of cells (unpublished data). We therefore limited all of our quantitative assays to colony sizes below 100 cells.

### Single-Gene Deletion Growth Rate Distributions

In each well of a 96-well plate, approximately equal cell numbers of a single-gene deletion strain and an easily distinguishable GFP fluorescent control strain, *FBA1*-GFP [64], were grown together for 9 h. The mean growth rate of the *FBA1*-GFP microcolonies was used to normalize deletion strain growth rates across different experimental wells. All reported single-gene deletion distributions are the combined microcolony growth rates from at least three replicate wells of a 96-well plate. Relative growth rates reported in Figures 1E and S3 and Table S1 were calculated by setting the mean growth rate of the control dubious open reading frame deletions (*YHR095W* and *YFR054C*) equal to one. Alternatively, growth rate distributions of deletions resulting in petite-negative and dubious open reading frame control strains (Figure S5) are reported as raw specific growth rates without normalization.

### Screen for Slow-Growth Markers

Using microarray analysis of cells grown in nutrient-limited chemostats, the regression slope for the transcriptional response to changes in growth rate has been determined for all transcripts [18]. The cell-to-cell variation, or noise, in protein level has been quantified for a large number of genes using flow cytometry of endogenously expressed GFP fusions and is summarized in a measure called DM [4]. To identify gene products that might mark cell-to-cell variation in growth rate, we set the following thresholds: a growth rate slope of less than −2 and noise (DM in synthetic dextrose medium) of greater than 5.

### Gene Ontology Enrichment

Gene ontology enrichment was calculated using the GO Term Finder in the Saccharomyces genome database website (http://www.yeastgenome.org/cgi-bin/GO/goTermFinder.pl) on September 7, 2011 using the default settings. Genes with a reported value for both growth rate slope and noise in synthetic dextrose medium were used as the set of background genes for statistical comparisons. Reported *p*-values are corrected for multiple hypothesis testing.

### *TSL1*-GFP Growth Rate Distributions

As is true for the single deletion studies, we observed only nominal differences between replicate wells or days (unpublished data). Thus, growth rate distributions and fluorescence correlation studies for the *TSL1*-GFP strain and associated controls were generated by pooling microcolony growth rates across a minimum of 12 replicate wells and two experimental days. Growth rate distributions associated with microscopy-based survival assays were performed by pooling microcolonies from a minimum of 80 replicate wells over a minimum of two experimental days.

### Heat Shock

Heat shock of film-covered glass-bottomed micro-well plates was performed by removing plates from the microscope and sandwiching them between two pre-heated standard aluminum heat blocks in a hydrated oven for 70 min at 60°C. Heat shock of *TSL1* and control knockout strains was performed in liquid suspension for 2 min at 60°C. Heat shock of sorted and unsorted *TSL1*-GFP strains was performed in liquid suspension for 6 min at 52°C.

### Growth-Rate Binned Survival

A *TSL1*Δ-mCherry control strain was constructed as a direct descendant of the *TSL1*-GFP strain (see “Yeast Strains and Cloning”). This genetic manipulation resulted in a mild but detectable decrease in mean population specific growth rate (0.366 h^−1^ for *TSL1*Δ-mCherry, 0.377 h^−1^ for *TSL1*-GFP, *p*<10^−10^, Wilcoxon-Mann-Whitney test). Because *TSL1* expression is generally low, requiring long exposure times and thus high fluorescence background, *TSL1*Δ-mCherry and *TSL1*-GFP were grown in a checkerboard pattern on separate wells on the same plate, rather than in the same well, to avoid genotype miscalls. We observed no obvious biases in growth rate or survival frequency over a plate's surface for either genotype and observed similar survival patterns over four similar heat-shock experiments.

### Multiple Logistic Regression

Heat-shock survival is a binary dependent variable, so multiple logistic regression was used to test the effects on it of growth rate (prior to heat shock) and genotype (*TSL1*-GFP vs. *TSL1*Δ-mCherry). A full linear model including main-effect terms for growth rate and genotype as well as an interaction between the two was compared to reduced models using AIC, as implemented in the glm and anova functions of R. The full model was not significantly better than a reduced model with the two main effects but without the interaction. Removing either main effect from this no-interaction model made the model significantly worse. Therefore reported *p*-values come from the model with both main effects but no interaction.

### Survival in Liquid Suspension

Heat shock of liquid suspensions was performed in triplicate. Survival frequency was determined by counting the number of cells in liquid before heat shock and comparing this to the number of colonies that grew on an agar plate following heat shock. *p*-Values were determined by performing a Student's *t* test on the arcsin of the square root of the proportion surviving.

### Cell Sorting

Cells were sonicated for 90 s in a Diagenode Bioruptor water bath prior to sorting. Cell sorting was performed on a FACSaria (BD) sorter. The pulse width was used to separate individual cells from cell clumps. FITC gates used to isolate cells with high levels of Tsl1-GFP are shown in Figure 4A. Because most cells contain low levels of Tsl1 (∼2,000 molecules per cell on average [66]), the sorter was not sensitive enough to sort cells in the bottom 85% of the distribution. Sorted cells were immediately resuspended in synthetic complete medium following sorting. A fraction of cells were immediately plated for growth rate distribution and heat shock survival analysis. A second fraction was grown in early- to mid-log phase for 48 h, allowed to reach saturation, grown again in early- to mid-log phase for 24 h, and plated for growth rate distribution analysis. Assuming an average specific growth rate of 0.4 h^−1^ for each sorted fraction, 76 h of log growth represents ∼42 generations.

### Replicative Age Analysis

*TSL1*-GFP cells were kept in logarithmic growth for a minimum of 24 h, sonicated for 90 s in a Diagenode Bioruptor water bath, washed once in PBS, fixed for 90 min in 3.7% formaldehyde, and washed twice in PBS. Bud scar staining was performed for 15 min in 1 mg ml^−1^ TRITC-labeled WGA. All sorting was done using a tight pulse-width gate to remove cell clumps from the analysis. For co-fluorescence measurements, ∼8×10^5^ cells were measured for WGA-TRITC and Tsl1-GFP fluorescence. Data shown are from one experiment. Replicate experiments yielded similar results. For bud scar counts, cells were sorted until 10^4^ cells were recovered. Cells were pelleted, resuspended in 5 µl Vectashield (Vector Laboratories), and mounted on a glass slide. Bud scars were counted manually using a Nikon TE2000e epifluorescent microscope and a 100× plan apochromat objective with a narrow focal plane. Three sorts for each category were performed and bud scars from ∼100 cells were counted per sort. Similar bud scar distributions were observed in all three sorts. Data shown are the pooled counts from all sorts.

## Supporting Information

Figure S1 Automated colony area measurements correlate with cell number. Manual cell counts are plotted against colony area measurements determined by automated image processing for cells binned by growth rate for the strain of the yeast deletion collection containing a knockout of *YFR054C*, an open reading frame with dubious function. (A) below two standard deviations (2 SD) from the mean population growth rate, (B) between 2 SD and 1 SD below the mean, (C) between 1 SD below the mean and 1 SD above the mean, (D) between 1 SD and 2 SD above the mean, (E) above 2 SD above the mean, (F) all counts from (A–E) plotted together. The purple line indicates the linear regression of the points.
(PDF)

Figure S2 Bland-Altman plot of automated and manual cell counts. Manual cell counts are plotted against the difference between cell count estimations on the basis of automated colony area measurements and manual cell counts. Cells are binned by growth rate for the strain of the yeast deletion collection containing a knockout of *YFR054C*, an open reading frame with dubious function. (A) below 2 SD from the mean population growth rate, (B) between 2 SD and 1 SD below the mean, (C) between 1 SD below the mean and 1 SD above the mean, (D) between 1 SD and 2 SD above the mean, (E) above 2 SD above the mean, (F) all counts from (A–E) plotted together. The purple line indicates the mean difference and orange lines indicate the 95% confidence interval.
(PDF)

Figure S3 Reproducibility of the microcolony growth rate assay. Eighteen replicate growth rate distributions are shown for six yeast strains. Traces of the same color are from replicate wells on the same microplate and traces of different colors are from replicate experimental days. In addition to the genotypes shown, each well contained an easily distinguishable fluorescent strain from the GFP fusion collection (*FBA1*-GFP, Invitrogen) [64] that was used to normalize growth rates for global differences between wells or experimental days (Materials and Methods). Thus, growth rates are reported as normalized values, with the mean *FBA1*-GFP growth rate within each well used as the normalizing denominator.
(PDF)

Figure S4 Effect of colony proximity on growth rate. Box plot of growth rates of colonies binned by the colony's proximity to its nearest neighbor for cells plated at a density of 2×10^4^ cells/ml. Colonies that fall within 35 µm (4–8 cell lengths) of their nearest neighbor do have reduced growth rates. This reduction may be due to local nutrient depletion or a technical problem with measuring the growth rates of closely spaced colonies (Materials and Methods). Regardless of the cause, we ignored all colonies with a nearest neighbor of less than 35 µm away in all our measurements. Whiskers are 1.5 times the interquartile range from the box.
(PDF)

Figure S5 Petite-negative strains contain slow-growing colonies. Cumulative specific growth rate distributions of six haploid knockout strains from the yeast deletion collection. Plotted are a control dubious open reading frame knockout (*YFR054C*, black) and five knockouts unable to grow when mitochondrial function is lost: *YME1* (red), *PET9* (green), *MGR1* (blue), *MGR2* (cyan), and *PDE2* (magenta).
(PDF)

Figure S6 Scatter plot of the means and standard deviations of the microcolony growth rates of 13 knockout strains from the yeast deletion collection. A linear regression (blue line) does not fit the data well.
(PDF)

Figure S7 Tsl1-GFP fluorescence intensity per unit area of colonies binned by growth rate for each time point during the first 6 h of growth. *p*-Values are a comparison to all colonies, Wilcoxon-Mann-Whitney test: *, *p*<0.01; **, *p*<1×10^−5^; ***, *p*<1×10^−10^.
(PDF)

Figure S8 Scatter plot of Tsl1-GFP fluorescence intensity per unit area and specific growth rates of microcolonies of cells expressing Tsl1-GFP under the endogenous *TSL1* promoter. A linear regression of the data is shown as a green line.
(PDF)

Figure S9 Fluorescence intensity per unit area of colonies binned by growth rate for *TSL1*-GFP (left) or *TMA108*-GFP cells (right). Error bars indicate SEM; *p*-values are a comparison to all colonies; Wilcoxon-Mann-Whitney test: **, *p*<1×10^−5^; ***, *p*<1×10^−10^.
(PDF)

Figure S10 Cumulative specific growth rate distributions of *TSL1*-GFP and *TSL1*Δ-mCherry microcolonies that contained at least one cell that survived heat killing or no surviving cells.
(PDF)

Table S1 Characteristics of growth rate distributions of single gene deletion strains.
(XLS)

Table S2 Candidate genes involved in bet hedging.
(XLS)

Table S3 Gene ontology enrichment of noisy gene products whose average expression correlates with the bulk growth rate.
(XLS)

Video S1 A typical field of microcolony growth. Cells in logarithmic growth are plated at a low density on a glass-bottomed multi-well plate and low-magnification time-lapse bright-field images are captured each hour (left). Custom-written software tracks colony area over time (right). Colonies that touch each other or the edge of the field discontinue being tracked. Notice an extremely slow-growing dark-purple colony at the lower left of the field.
(MOV)

Video S2 A video of the same colonies shown in Figure 1A.
(MOV)

Video S3 A highly fluorescent slow-growing cell produces low-fluorescence fast-growing progeny. Bright-field (bottom) and fluorescent (top) images of *TSL1*-GFP yeast grown for 9 h. Notice that slow-growing cells on the right produce fast-growing progeny.
(MOV)

Video S4 Typical results of heat killing. Bright-field (left) and fluorescent (right) images of *TSL1*-GFP yeast grown for 5 h, heat shocked, and grown for another 13 h.
(MOV)

Video S5 A second field, as described in Video S4.
(MOV)

## Abbreviations

AER: age expression ratio
GFP: green fluorescent protein
PFS: perfect focus system
SD: standard deviation
SEM: standard error of the mean
WGA-TRITC: wheat-germ agglutinin tetramethyl rhodamine isothiocyanate
