# Supplementary figures and images for: Bet Hedging in Yeast by Heterogeneous, Age-Correlated Expression of a Stress Protectant
Source: PLoS Biol. 2012 May 8;10(5):e1001325. doi: 10.1371/journal.pbio.1001325 (PMC3348152; doi:10.1371/journal.pbio.1001325)

**A**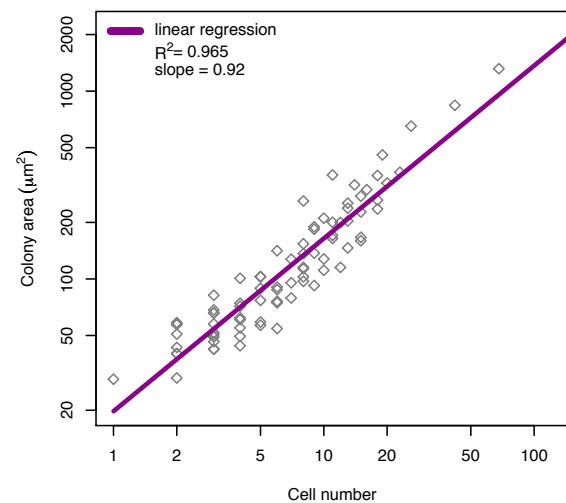**B**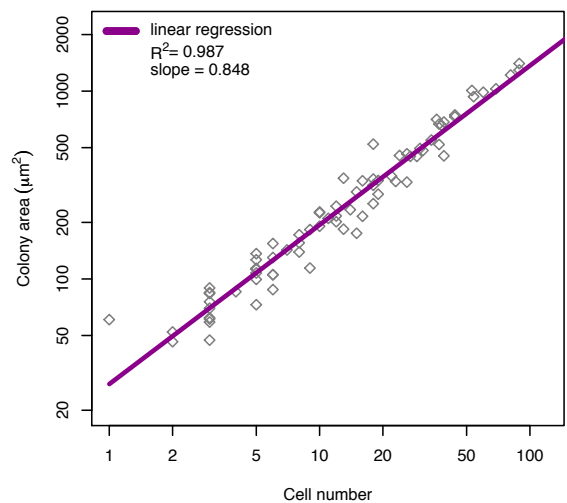**C**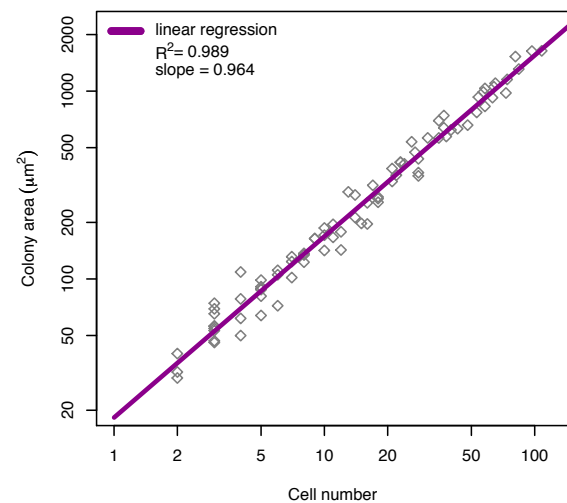**D**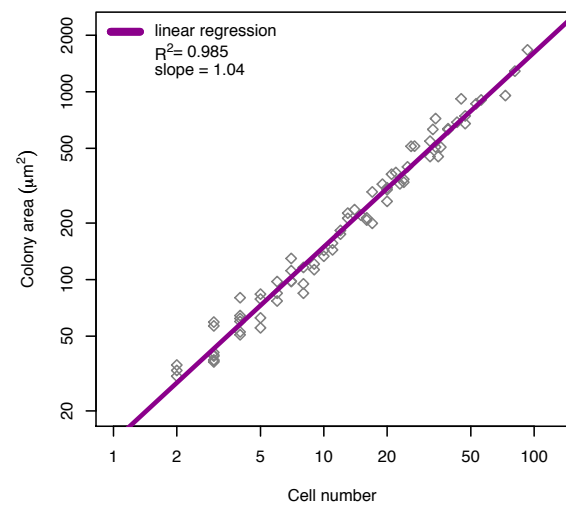**E**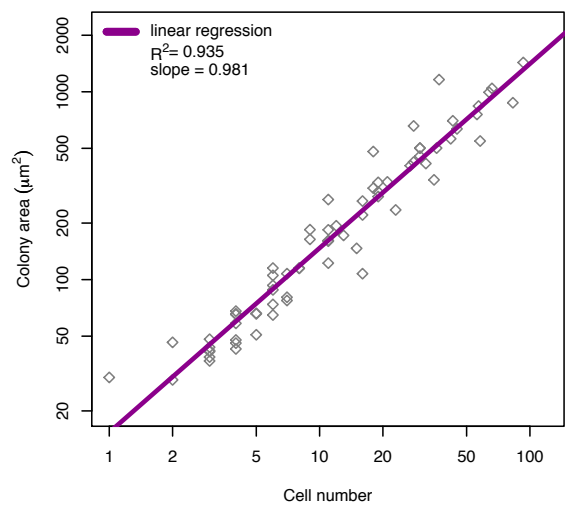**F**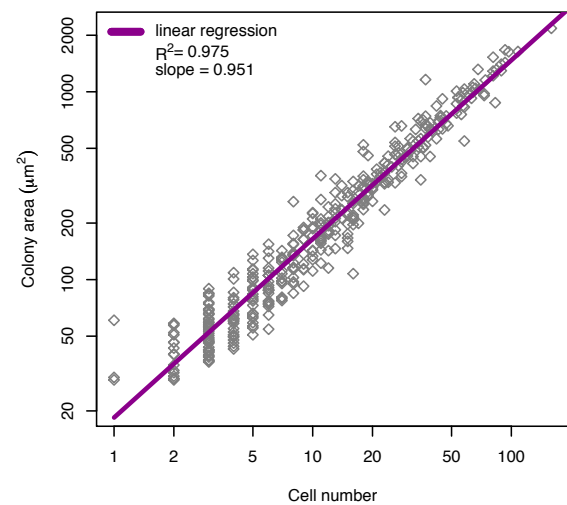

Supplement: Figure S1 — Automated colony area measurements correlate with cell number. Manual cell counts are plotted against colony area measurements determined by automated image processing for cells binned by growth rate for the strain of the yeast deletion collection containing a knockout of YFR054C, an open reading frame with dubious function. (A) below two standard deviations (2 SD) from the mean population growth rate, (B) between 2 SD and 1 SD below the mean, (C) between 1 SD below the mean and 1 SD above the mean, (D) between 1 SD and 2 SD above the mean, (E) above 2 SD above the mean, (F) all counts from (A–E) plotted together. The purple line indicates the linear regression of the points. (PDF) [file pbio.1001325.s001.pdf]

**A**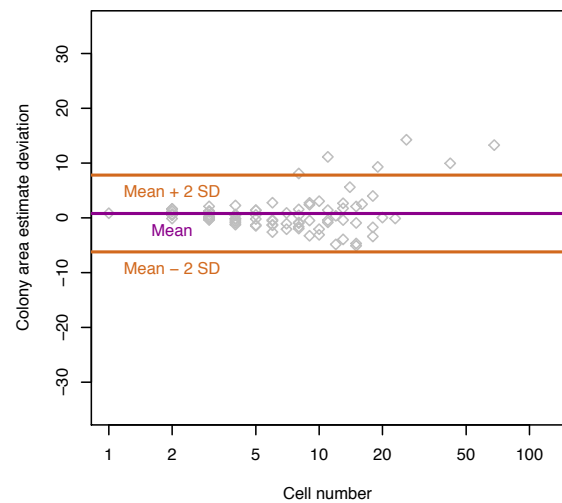**B**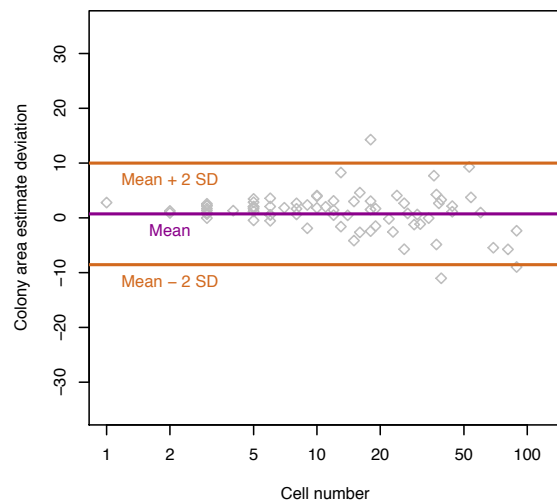**C**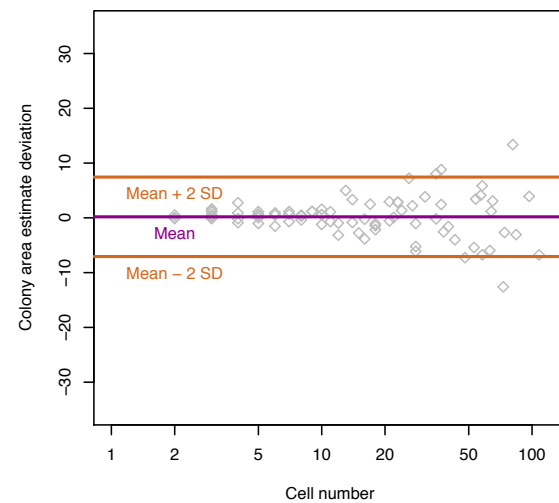**D**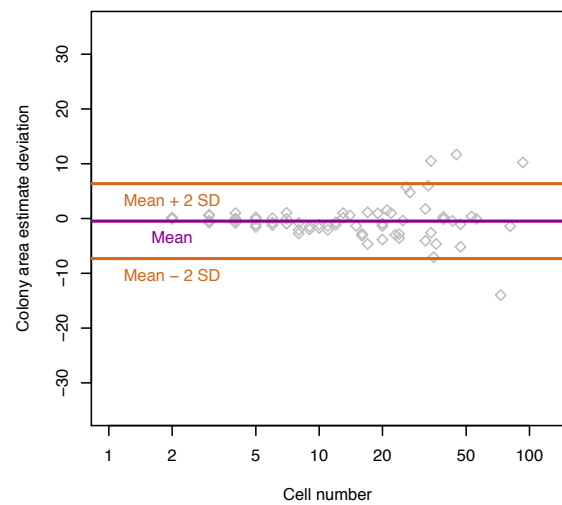**E**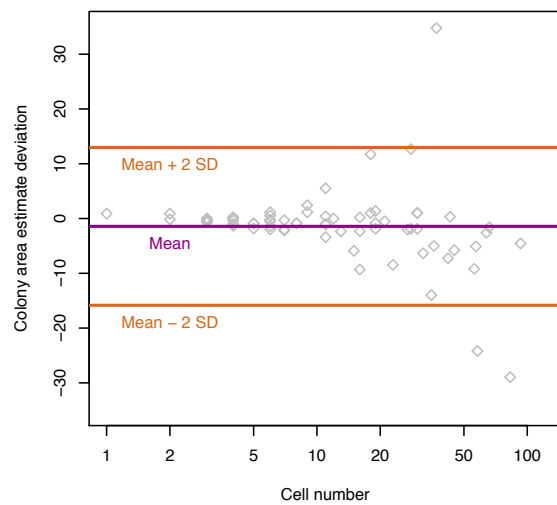**F**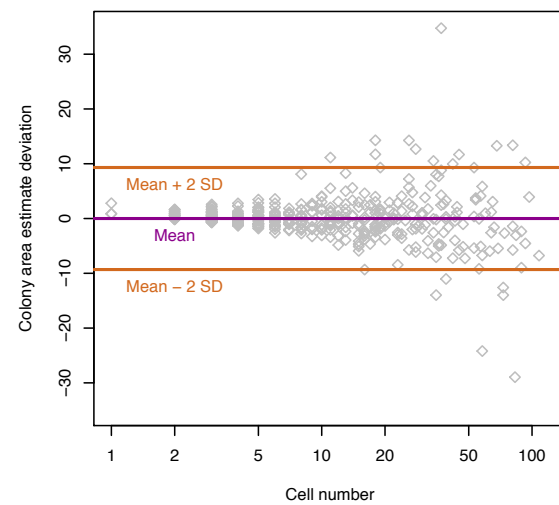

Supplement: Figure S2 — Bland-Altman plot of automated and manual cell counts. Manual cell counts are plotted against the difference between cell count estimations on the basis of automated colony area measurements and manual cell counts. Cells are binned by growth rate for the strain of the yeast deletion collection containing a knockout of YFR054C, an open reading frame with dubious function. (A) below 2 SD from the mean population growth rate, (B) between 2 SD and 1 SD below the mean, (C) between 1 SD below the mean and 1 SD above the mean, (D) between 1 SD and 2 SD above the mean, (E) above 2 SD above the mean, (F) all counts from (A–E) plotted together. The purple line indicates the mean difference and orange lines indicate the 95% confidence interval. (PDF) [file pbio.1001325.s002.pdf]

**YFR054C**

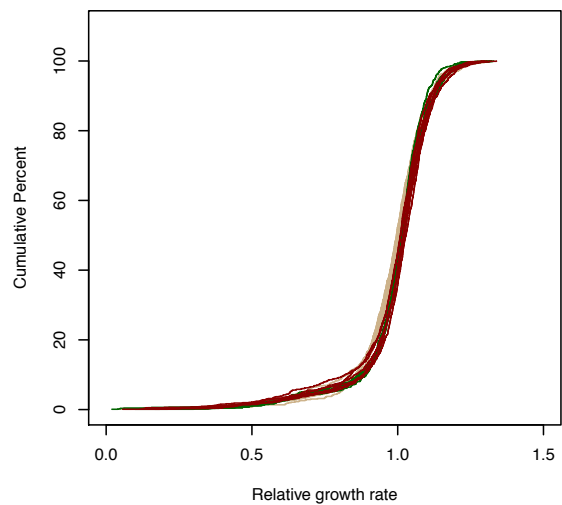

**PET9**

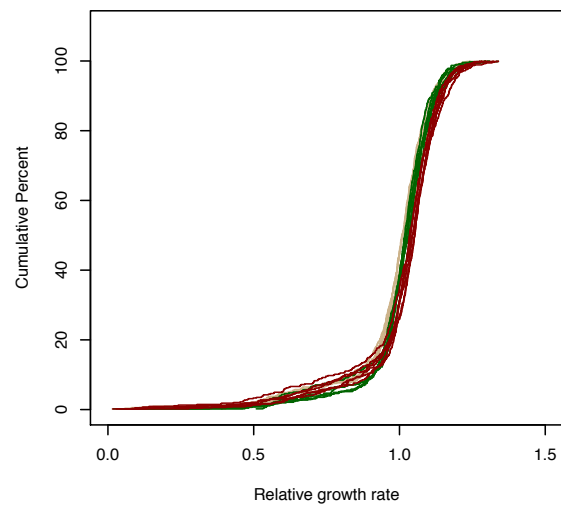

**YME1**

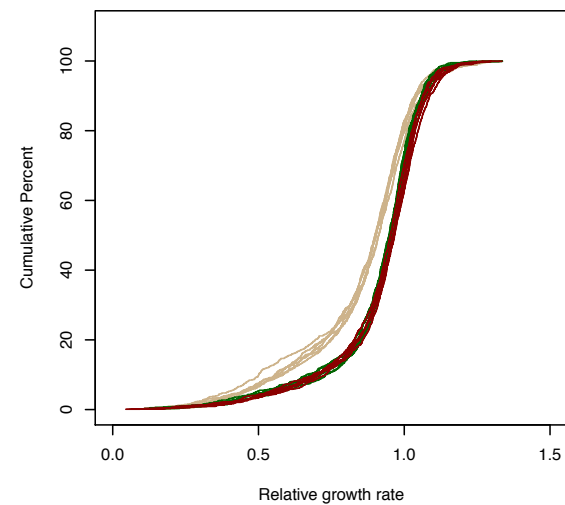

**HTZ1**

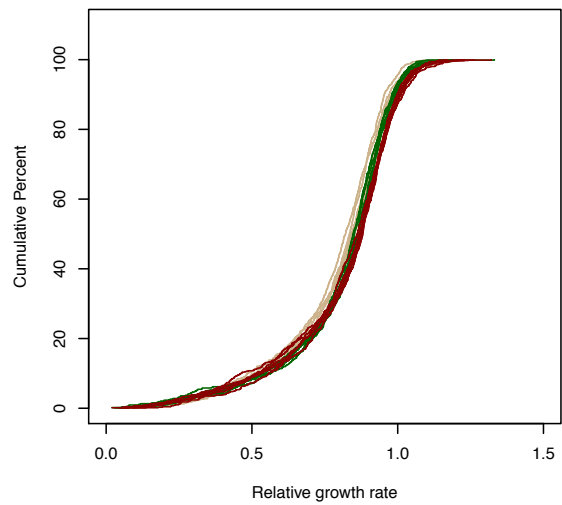

**RAD50**

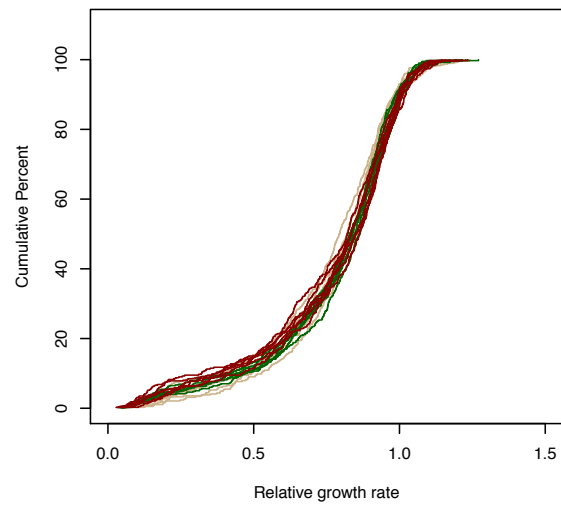

**SNF6**

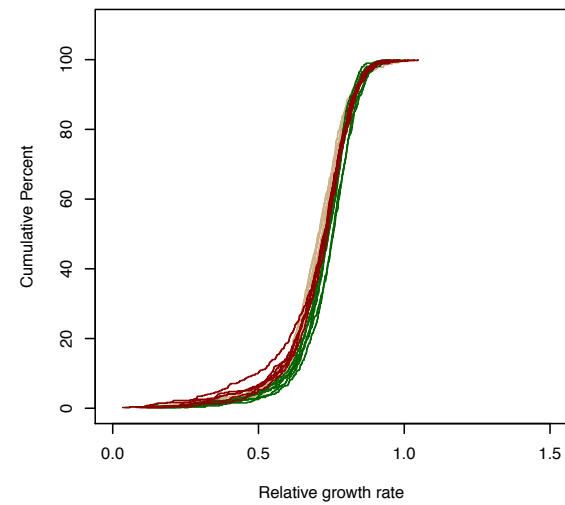

Supplement: Figure S3 — Reproducibility of the microcolony growth rate assay. Eighteen replicate growth rate distributions are shown for six yeast strains. Traces of the same color are from replicate wells on the same microplate and traces of different colors are from replicate experimental days. In addition to the genotypes shown, each well contained an easily distinguishable fluorescent strain from the GFP fusion collection (FBA1-GFP, Invitrogen) [64] that was used to normalize growth rates for global differences between wells or experimental days (Materials and Methods). Thus, growth rates are reported as normalized values, with the mean FBA1-GFP growth rate within each well used as the normalizing denominator. (PDF) [file pbio.1001325.s003.pdf]

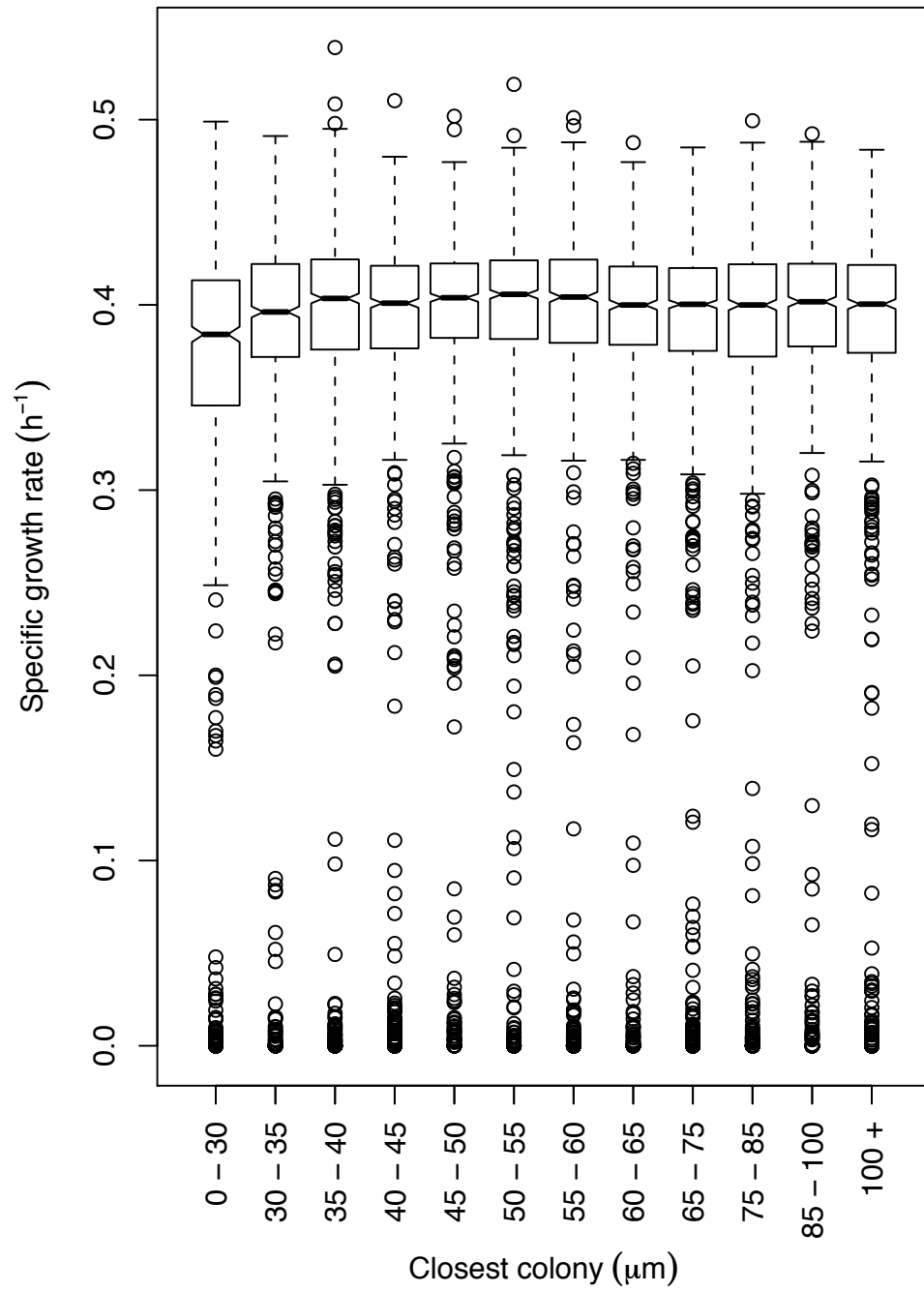

Supplement: Figure S4 — Effect of colony proximity on growth rate. Box plot of growth rates of colonies binned by the colony's proximity to its nearest neighbor for cells plated at a density of 2×10^4^ cells/ml. Colonies that fall within 35 µm (4–8 cell lengths) of their nearest neighbor do have reduced growth rates. This reduction may be due to local nutrient depletion or a technical problem with measuring the growth rates of closely spaced colonies (Materials and Methods). Regardless of the cause, we ignored all colonies with a nearest neighbor of less than 35 µm away in all our measurements. Whiskers are 1.5 times the interquartile range from the box. (PDF) [file pbio.1001325.s004.pdf]

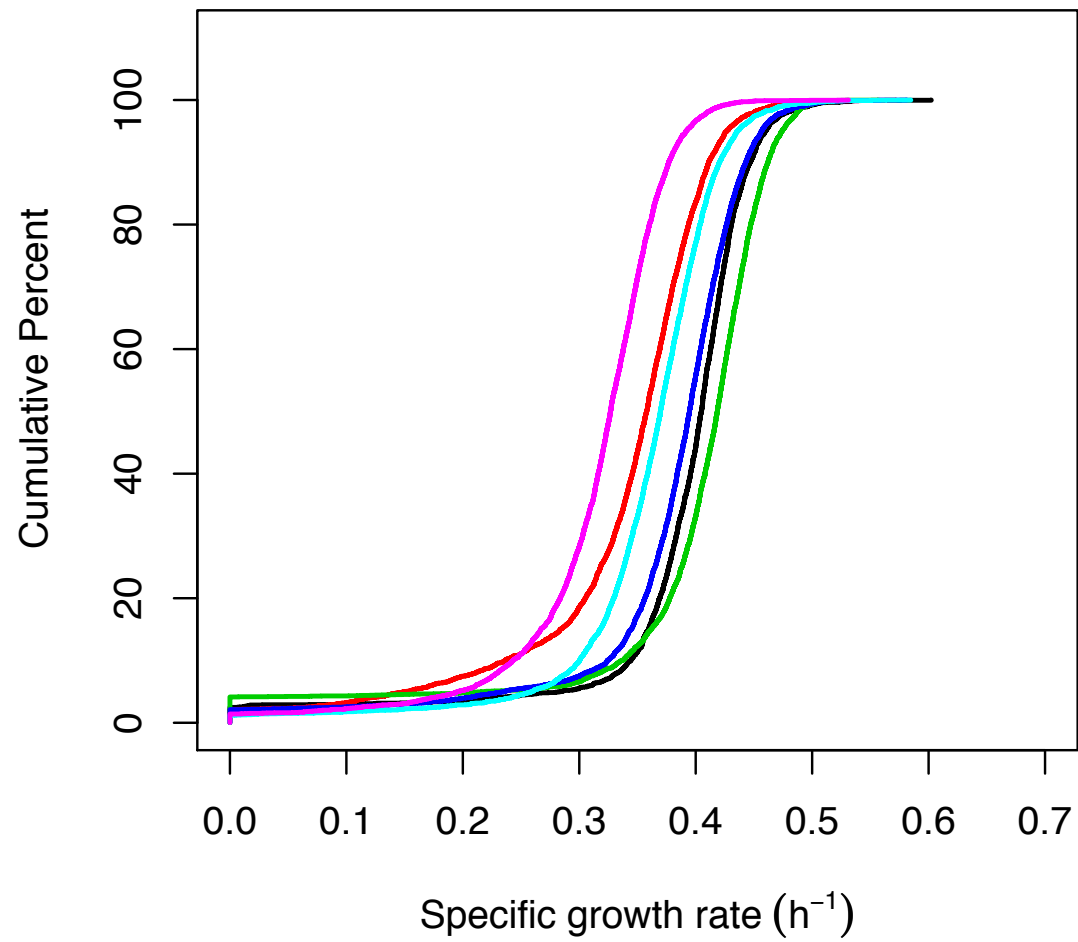

Supplement: Figure S5 — Petite-negative strains contain slow-growing colonies. Cumulative specific growth rate distributions of six haploid knockout strains from the yeast deletion collection. Plotted are a control dubious open reading frame knockout (YFR054C, black) and five knockouts unable to grow when mitochondrial function is lost: YME1 (red), PET9 (green), MGR1 (blue), MGR2 (cyan), and PDE2 (magenta). (PDF) [file pbio.1001325.s005.pdf]

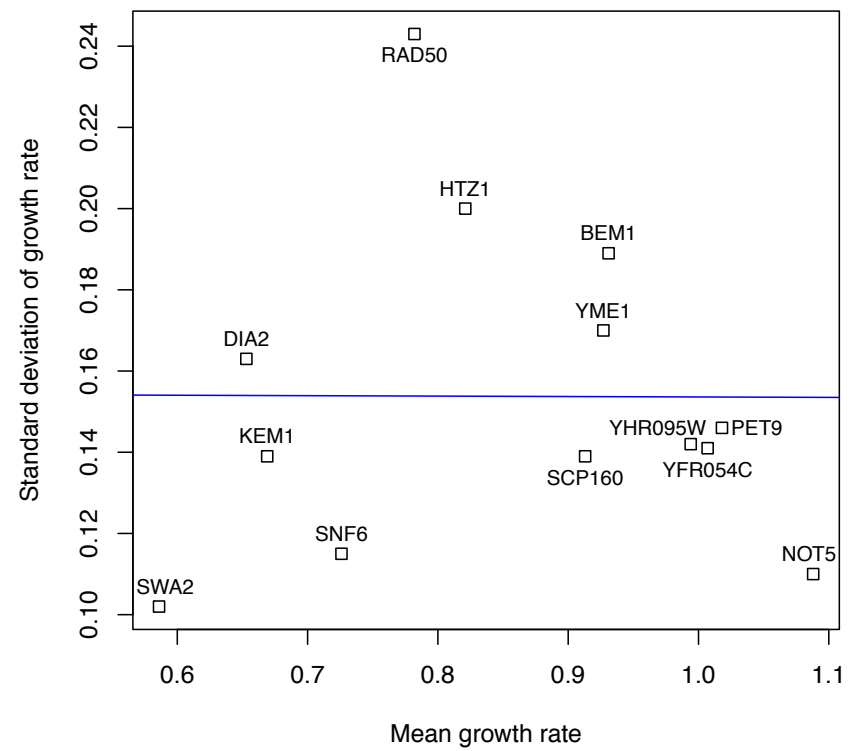

Supplement: Figure S6 — Scatter plot of the means and standard deviations of the microcolony growth rates of 13 knockout strains from the yeast deletion collection. A linear regression (blue line) does not fit the data well. (PDF) [file pbio.1001325.s006.pdf]

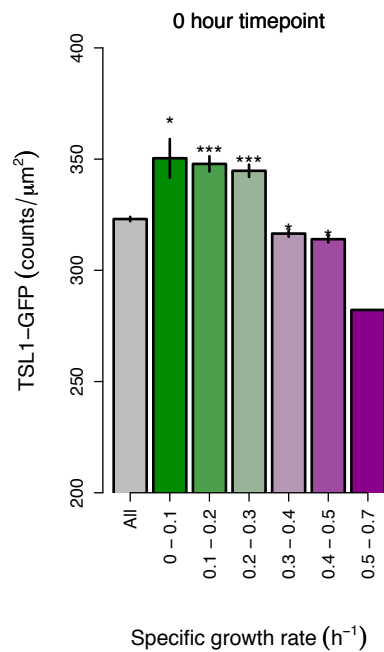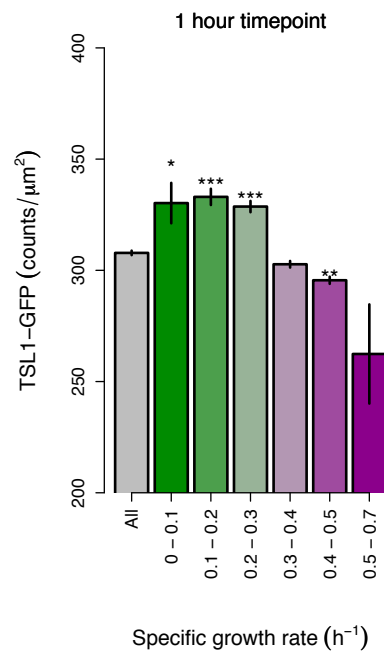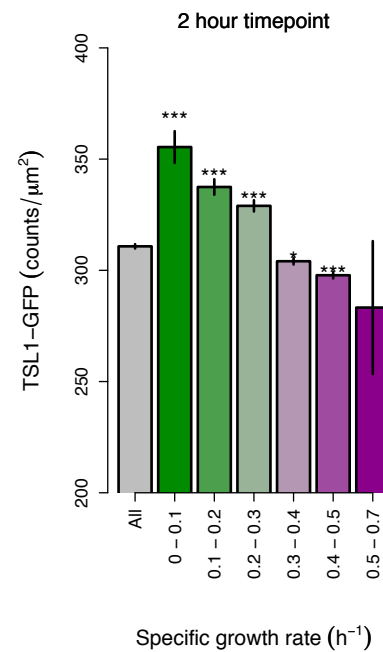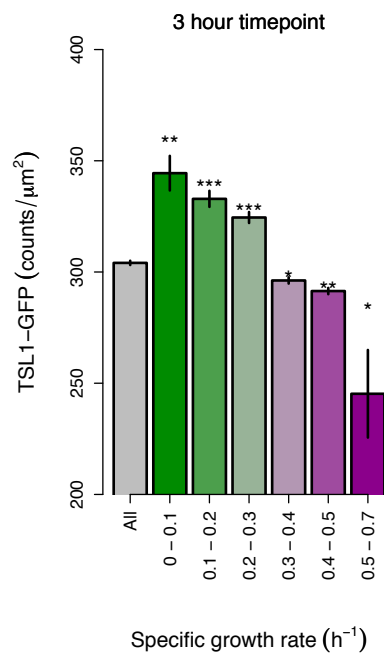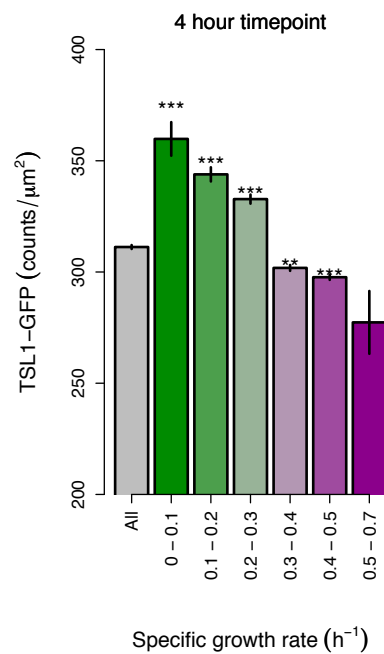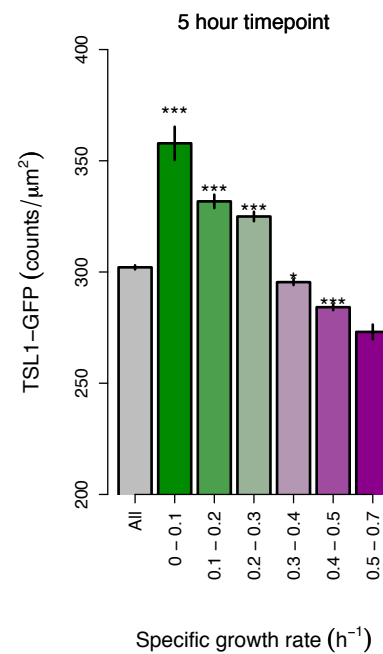

Supplement: Figure S7 — Tsl1-GFP fluorescence intensity per unit area of colonies binned by growth rate for each time point during the first 6 h of growth. p-Values are a comparison to all colonies, Wilcoxon-Mann-Whitney test: *, p<0.01; **, p<1×10^−5^; ***, p<1×10^−10^. (PDF) [file pbio.1001325.s007.pdf]

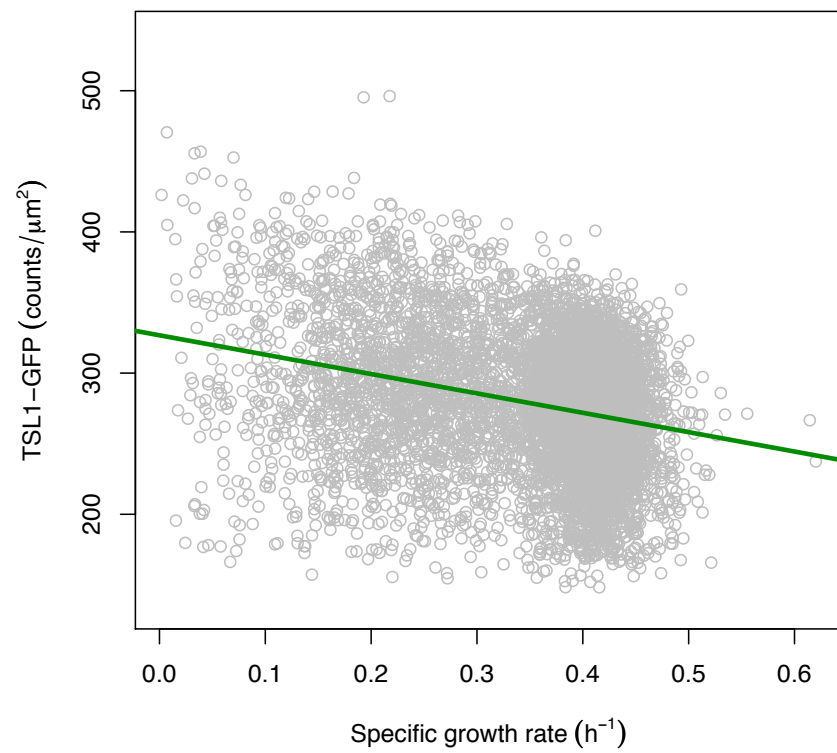

Supplement: Figure S8 — Scatter plot of Tsl1-GFP fluorescence intensity per unit area and specific growth rates of microcolonies of cells expressing Tsl1-GFP under the endogenous TSL1 promoter. A linear regression of the data is shown as a green line. (PDF) [file pbio.1001325.s008.pdf]

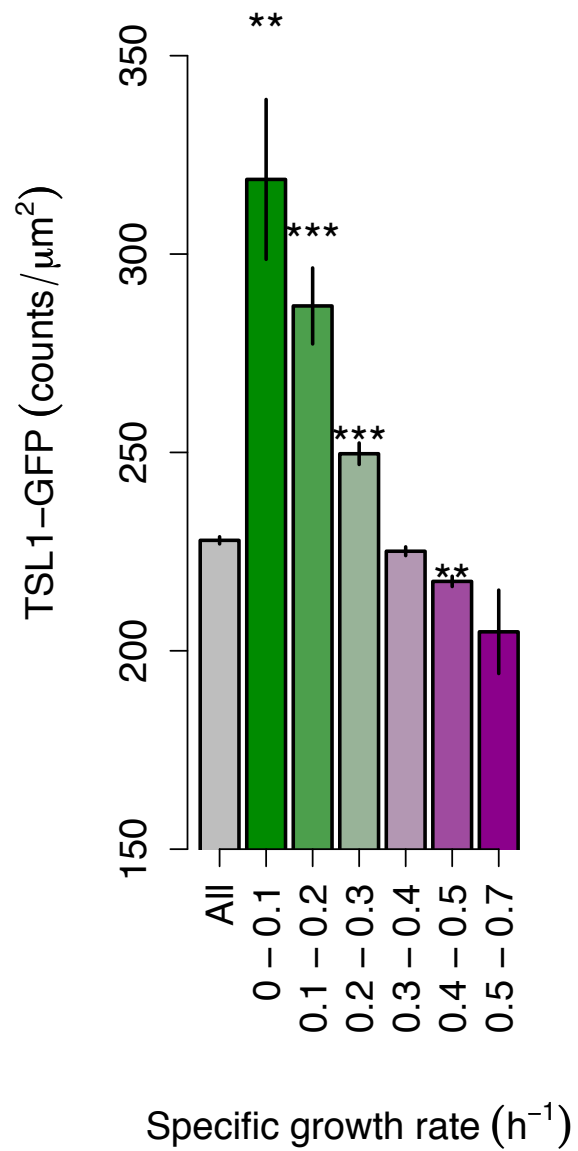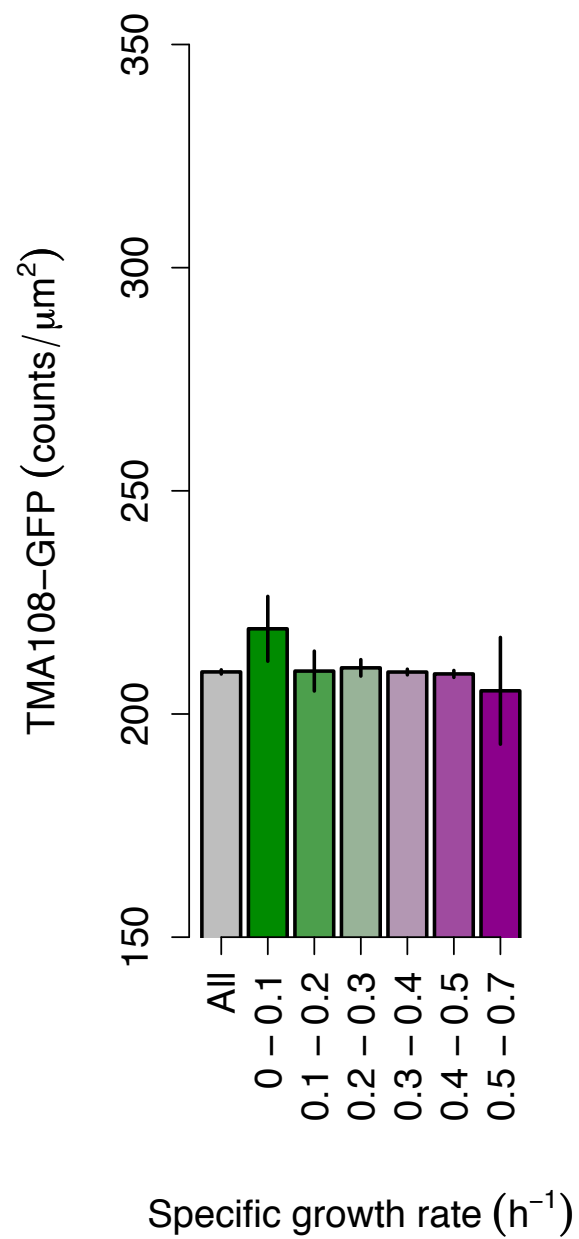

Supplement: Figure S9 — Fluorescence intensity per unit area of colonies binned by growth rate for TSL1-GFP (left) or TMA108-GFP cells (right). Error bars indicate SEM; p-values are a comparison to all colonies; Wilcoxon-Mann-Whitney test: **, p<1×10^−5^; ***, p<1×10^−10^. (PDF) [file pbio.1001325.s009.pdf]

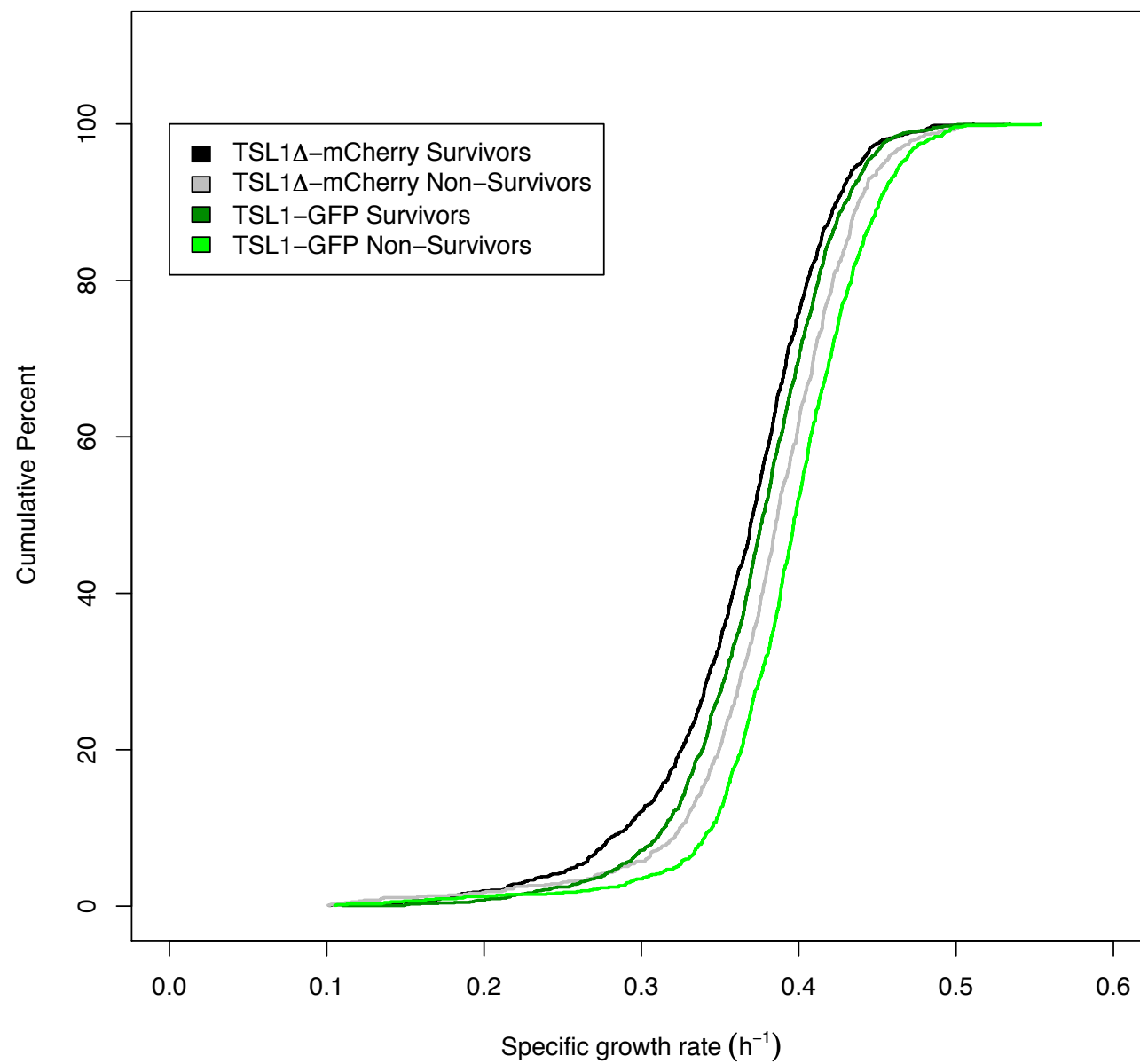

Supplement: Figure S10 — Cumulative specific growth rate distributions of TSL1-GFP and TSL1Δ-mCherry microcolonies that contained at least one cell that survived heat killing or no surviving cells. (PDF) [file pbio.1001325.s010.pdf]
